# Noise sensitivity of ^89^Zr-Immuno-PET radiomics based on count-reduced clinical images

**DOI:** 10.1186/s40658-022-00444-4

**Published:** 2022-03-03

**Authors:** Ananthi Somasundaram, David Vállez García, Elisabeth Pfaehler, Yvonne W. S. Jauw, Josée M. Zijlstra, Guus A. M. S. van Dongen, Willemien C. Menke-van der Houven van Oordt, Marc C. Huisman, Elisabeth G. E. de Vries, Ronald Boellaard

**Affiliations:** 1 Department of Nuclear Medicine and Molecular Imaging, University Medical Center Groningen, Hanzeplein 1, 9713 GZ Groningen, The Netherlands; 2 Department of Radiology and Nuclear Medicine, Amsterdam UMC - Location VU University Medical Center, Amsterdam, The Netherlands; 3 Department of Nuclear Medicine, University Hospital Augsburg, Augsburg, Germany; 4 Department of Hematology, Amsterdam UMC - Location VU University Medical Center, Amsterdam, The Netherlands; 5 Department of Medical Oncology, Amsterdam UMC - Location VU University Medical Center, Amsterdam, The Netherlands; 6 Department of Medical Oncology, University Medical Center Groningen, University of Groningen, Groningen, The Netherlands

**Keywords:** ^89^Zr-Immuno PET, Radiomics, Noise, Precision, Bias, Repeatability, Reproducibility

## Abstract

**Purpose:**

Low photon count in ^89^Zr-Immuno-PET results in images with a low signal-to-noise ratio (SNR). Since PET radiomics are sensitive to noise, this study focuses on the impact of noise on radiomic features from ^89^Zr-Immuno-PET clinical images. We hypothesise that ^89^Zr-Immuno-PET derived radiomic features have: (1) noise-induced variability affecting their precision and (2) noise-induced bias affecting their accuracy. This study aims to identify those features that are not or only minimally affected by noise in terms of precision and accuracy.

**Methods:**

Count-split ^89^Zr-Immuno-PET patient scans from previous studies with three different ^89^Zr-labelled monoclonal antibodies were used to extract radiomic features at 50% (S50p) and 25% (S25p) of their original counts. Tumour lesions were manually delineated on the original full-count ^89^Zr-Immuno-PET scans. Noise-induced variability and bias were assessed using intraclass correlation coefficient (ICC) and similarity distance metric (SDM), respectively. Based on the ICC and SDM values, the radiomic features were categorised as having poor [0, 0.5), moderate [0.5, 0.75), good [0.75, 0.9), or excellent [0.9, 1] precision and accuracy. The number of features classified into these categories was compared between the S50p and S25p images using Fisher’s exact test. All *p* values < 0.01 were considered statistically significant.

**Results:**

For S50p, a total of 92% and 90% features were classified as having good or excellent ICC and SDM respectively, while for S25p, these decreased to 81% and 31%. In total, 148 features (31%) showed robustness to noise with good or moderate ICC and SDM in both S50p and S25p. The number of features classified into the four ICC and SDM categories between S50p and S25p was significantly different statistically.

**Conclusion:**

Several radiomic features derived from low SNR ^89^Zr-Immuno-PET images exhibit noise-induced variability and/or bias. However, 196 features (43%) that show minimal noise-induced variability and bias in S50p images have been identified. These features are less affected by noise and are, therefore, suitable candidates to be further studied as prognostic and predictive quantitative biomarkers in ^89^Zr-Immuno-PET studies.

**Supplementary Information:**

The online version contains supplementary material available at 10.1186/s40658-022-00444-4.

## Introduction

Treating patients with cancer with monoclonal antibodies (mAbs) has been proven beneficial [1]. However, not all patients benefit and these treatments can induce side effects and are expensive. Recent studies have shown that inter- and intra-tumour heterogeneity can contribute to treatment failure and, hence, it can affect treatment decisions that are generally based on single tumour biopsy [2]. Positron emission tomography (PET) imaging with ^89^Zr-labelled mAbs, commonly referred to as ^89^Zr-Immuno-PET, was able to predict the efficacy of immunotherapy in small studies [3–5]. Therefore, analysing ^89^Zr-Immuno-PET more extensively is of interest. PET/CT standardised uptake values (SUV_peak_, SUV_mean_, and SUV_max_) and other standard semi-quantitative metrics, such as metabolically active tumour volume (MATV), are based on one voxel or the average over all the voxels within the volume of interest (VOI) and, hence, do not fully capture all aspects of tumour uptake characteristics.

The aforementioned heterogeneity in tumour uptake characteristics can be quantified with radiomics, which is the high throughput extraction of quantitative features from medical images [6–8]. These features are divided into families based on, for example, morphology, local intensity, intensity-based statistics, intensity histogram, and different texture matrices. Radiomics may have the potential to support personalised immunotherapy, for example, by helping to identify patients who might benefit from a specific treatment and to identify the treatment that can help a specific patient group [6–8]. To our best knowledge, the clinical value of ^89^Zr-Immuno-PET derived radiomics have not yet been explored and, so far, mainly standard PET uptake metrics were used [5]. However, before the clinical value of radiomics can be studied, it is important to understand the sources of error and uncertainties of these radiomic features, particularly under the low count conditions of ^89^Zr-Immuno-PET studies.

^89^Zr-Immuno-PET suffers from low photon counts for two reasons. Firstly, ^89^Zr has a low positron yield (22.6%). Secondly, to keep the radiation exposure within acceptable or legal levels, the amount of injected activity is required to be low (e.g. 37 MBq for cancer immunotherapy or 18 MBq for rheumatoid arthritis [9]) because of the long half-life of ^89^Zr (78.4 h). This low photon count results in images with a rather poor signal-to-noise ratio (SNR). This low SNR has been shown to affect the SUV measurements in these images [9–11]. At the same time, PET radiomic features are also sensitive to noise [12–14]. For quantitative metrics, it is important to be repeatable, reproducible, and reliable. In ^89^Zr-Immuno-PET studies, it is necessary to explore the influence of noise on the bias and precision of the radiomic features.

This study, therefore, focusses on the impact of noise on the radiomic features extracted from ^89^Zr-Immuno PET clinical images. Jauw et al*.* assessed the noise-induced variability and reliability of SUV measurements using repeatability coefficients (RC) and intraclass correlation coefficient (ICC) in count-split ^89^Zr-Immuno-PET clinical images [9]. The same dataset has been used in our study to assess the bias and precision of radiomic features. We hypothesise that ^89^Zr-Immuno-PET derived radiomic features will have: (1) noise-induced variability affecting their precision and (2) noise-induced bias affecting their accuracy. This study aims to identify those features that are not or only minimally affected by noise in terms of precision and accuracy.

## Materials and methods

### Dataset

^89^Zr-Immuno-PET scans (*n* = 20) with low-dose CT from previous studies with three different ^89^Zr-labelled mAbs were used: ^89^Zr-antiCD20 mAb (*n* = 6), ^89^Zr-anti-epidermal growth factor receptor (EGFR) mAb (*n* = 3), and ^89^Zr-antiCD44 mAb (*n* = 11), used for the treatment of patients with non-Hodgkin lymphoma, colorectal cancer, and in an all-comer phase 1 clinical trial for solid tumours, respectively [15–17]. The injected activity and activity at scan start were 74 MBq (73.55 ± 0.43 MBq) and 20 MBq (20.44 ± 0.62 MBq) for ^89^Zr-antiCD20 mAb, 37 MBq (36.12 ± 0.17 MBq) and 10 MBq (10.02 ± 0.1 MBq) for ^89^Zr-anti-EGFR, and 37 MBq (36.58 ± 0.22 MBq) and 15 MBq (15.41 ± 0.7 MBq) for ^89^Zr-antiCD44 mAb. All scans were acquired using Philips GEMINI 64 or Ingenuity PET/CT scanners for 5 min per bed position. The images were reconstructed using European Association of Nuclear Medicine Research Ltd (EARL1) compliant settings [18]: 3D BLOB-OS-TF method (3 iterations, 33 subsets) with a matrix size of 144 × 144 and a voxel size of 4 × 4 × 4 mm. Further study procedures, including image acquisition and reconstruction protocols, have been reported before in detail [9, 15–17]. Patients were originally scanned on different days after tracer injection. Only their last day scan was used for an optimal contrast-to-noise ratio setting: day 6 for ^89^Zr-antiCD20 mAb and ^89^Zr-anti-EGFR mAb, and day 4 for ^89^Zr-antiCD44 mAb. In one out of the 20 patients, the day 6 scan was not available. This patient was scanned with ^89^Zr-antiCD20 mAb (P20) and excluded from the analysis. Scans with similar activity at scan start (13–22 MBq) were aimed at, so that they have statistically similar image quality. Therefore, the scans of three patients (P07, P18, P19) with low activity at scan start (< 13 MBq) were excluded from the analysis. Of the remaining 16 patients, nine had specific tracer uptake in the tumour lesions. These nine patients with a total of 47 tumour lesions were, therefore, included for tumour radiomic analysis. The interquartile range of tumour SUVpeak and volume was 4.0–12.6 and 2.8–14.8 ml, respectively, with corresponding median values of 6.1 and 6.3 ml (Additional file 1: Fig. S1). Additionally, all 16 patients were included for the extraction of radiomic metrics on normal background tissue (hereafter referred to as BG). The number of tumour lesions per patient, along with other relevant clinical and patient demographic data, is listed in Additional file 1: Table S1.

In this dataset, count-split PET list-mode data were used to assess precision and bias of ^89^Zr-Immuno-PET radiomic features at 50% and 25% of the original counts, hereafter referred to as S50p and S25p, respectively. To this end, the alternate counts in the raw full-count PET list-mode data were separated apart so that two equal data sets were created. Then, the split list-mode data was individually reconstructed into two count-reduced images. Each of these two count-reduced images was considered to be statistically independent of each other, as they would have been obtained with 50% of the original injected activity under identical scan conditions. Hence, the variability in the two count-split images can be considered to be due to noise only. The S50p list-mode data were then split into two and reconstructed again, resulting in new images (S25p) similar to those which would have been obtained with 25% of the injected activity. Further details of the count-split procedure of PET list-mode data along with the scheme can be found elsewhere [9]. Example images of an ^89^Zr-Immuno-PET patient scan reconstructed with original 100%, 50% (S50p) and 25% (S25p) counts are shown in Additional file 1: Fig. S2.

### Segmentation

Tumour lesions were manually delineated on the original full-count ^89^Zr-Immuno-PET scans by a nuclear medicine physician using ACCURATE tool [19], with the low dose CT for anatomical reference [9].

VOIs were defined manually in BG tissues using spheres with a fixed diameter of 3 cm for the liver, spleen, muscle, brain, and lung, and 2 cm for the kidney, and using multiple fixed-size circular ROIs of 2.0 and/or 1.5 cm in successive slices of the aortic arch to estimate the blood pool activity concentration.

### Radiomic feature calculation

A total of 458 radiomic features, belonging to ten feature groups, were extracted using the RaCaT tool [20] (version 1.19) as per the Imaging Biomarker Standardisation Initiative (IBSI) guidelines [21]. (Table 1 shows the feature groups with the number of radiomic features per group.) Morphological features were excluded from the analysis, as they only depend on the segmentation and not on the image quality of the PET data. All the remaining features are listed in Additional file 1: Table S5. Before radiomic feature calculation, the images were converted from Bq/ml to SUV by normalisation using the whole-body weight. This continuous SUV intensity scale was then discretised using a fixed bin width (FBW) of 0.25 SUV for calculating the texture features. FBW discretisation has been found to give more repeatable radiomic features than the alternative method of fixed bin number (FBN) [12]. Textural features were calculated with an isotropic voxel size of 2 mm, using resampling and linear interpolation of the images and VOIs as recommended [13]. A 26-connected neighbourhood in 3D and an 8-connected neighbourhood in 2D were used to analyse the distribution of voxels, with the neighbourhood consisting of voxels within Chebyshev distance of 1.

**Table 1:** Radiomic feature groups with the number of robust (excellent ICC and SDM) features in S50p and S25p images

Radiomic feature group	# Total radiomic features	# Robust features in S50p	# Robust features in S25p
Local intensity	2	2	0
Statistics	18	5	2
Intensity histogram	24	5	2
Intensity volume histogram (IVH)	6	1	0
Grey level co-occurrence (GLCM)	150	52	12
Grey level run length (GLRLM)	96	60	6
Grey level size zone (GLSZM)	48	17	2
Grey level distance zone (GLDZM)	48	27	9
Neighbourhood grey tone difference (NGTDM)	15	4	0
Neighbouring grey-level dependence (NGLDM)	51	23	0
Total	458	196	33

### Analysis of radiomic features

All subsequent data analysis was performed using Python (version 3.7.6). Analysis of precision and noise-induced bias were performed separately for tumour and BG VOI.

A different number of tumour lesions per patient might cause a bias in the calculations of precision and accuracy metrics. Therefore, a subsampling technique based on random sampling without replacement was used with 100 iterations, where the same tumour sample cannot be drawn more than once at each iteration, and a maximum of three tumour lesions were drawn per patient at each iteration. This procedure was performed to avoid data imbalance during the assessment of the variability metrics. For the final metric, the mean and standard deviation (SD) of the metrics from all iterations was calculated along with the 95% confidence intervals (CI) using the percentile method.

#### Analysis of precision

The precision of the radiomic features was analysed using the intraclass correlation coefficient (ICC). A two-way mixed-effects model was used to evaluate the absolute agreement between the radiomic features derived from the statistically independent count-reduced images. The ICC estimates were calculated using the R package: irr [22] (version 0.84.1) and the rpy2 package [23] (version 2.9.4) as the Python interface.

Based on the mean ICC values after subsampling, the radiomic features were categorised as having poor (ICC < 0.5), moderate (0.5 ≤ ICC < 0.75), good (0.75 ≤ ICC < 0.9), or excellent (ICC ≥ 0.9) precision.

To study the effect of noise on radiomic feature performance, the percentage of features belonging to each of the four categories was calculated per feature group and in total for S50p and S25p images.

#### Analysis of noise-induced bias

As a measure of accuracy, a similarity distance metric (SDM) was used to quantify the noise-induced bias between the radiomic features derived from the count-split images (i.e. S50p and S25p images) and the full-count images. This metric was calculated as the ratio of the variance between the tumour features in the full-count images to the total variance, where the total variance is the sum of variance between the tumour features in the full-count images and squared Euclidean distance between the full-count and the count-split feature values. Thus, if the calculated feature has a low noise-induced bias for count-split images with a high noise level, the squared Euclidean distance between the full-count and the count-split image features is negligible when compared to the differences in feature values seen between the different tumour lesions. SDM values range from 0 (low accuracy or large noise-induced bias) to 1 (high accuracy or negligible noise-induced bias).

SDM was calculated using the formula:

$$\mathrm{SDM}= \frac{1}{m}\sum_{1}^{m}\frac{{\sigma }_{f}^{2}}{(\frac{1}{n} \sum_{1}^{n}{d}^{2}\left(f,r\right))+ {\sigma }_{f}^{2}}$$

where *n* is the number of tumour lesions, *m* is the number of repeated measurements (i.e. 2 for S50p and 4 for S25p), $${\sigma }_{f}^{2}$$ is the variance between features of *n* tumour lesions in the full-count scan ($$f)$$, $${d}^{2}\left(f,r\right)$$ is the squared Euclidean distance between the full-count ($$f)$$ and count-reduced scan feature ($$r$$) values.

Based on the mean SDM values after subsampling, the radiomic features were categorised as having poor (SDM < 0.5), moderate (0.5 ≤ SDM < 0.75), good (0.75 ≤ SDM < 0.9), or excellent (SDM ≥ 0.9) accuracy. In addition, the percentage of features belonging to each of the four categories was calculated per feature group and in total for S50p and S25p images.

#### Statistical analysis

In order to check if the noise level in the images affects categorization of radiomics based on ICC and SDM, the number of features classified into the four ICC and SDM categories was compared between the S50p and S25p images using Fisher’s exact test. Fisher’s exact test was chosen because it has higher power and is exact compared to Chi-square test which is only approximate and not exact in the presence of small samples. All p-values below 0.01 were considered statistically significant. A more restrictive threshold for p-value was chosen instead of the standard threshold of 0.05 to take into account the multiple feature groups. Fisher's exact test was performed using the R package stats [24] (version 3.6.2) and the rpy2 package [23] (version 2.9.4) as the Python interface.

## Results

### Analysis of precision (ICC)

#### Analysis of precision (ICC) in tumour lesions

**Fig. 1:**
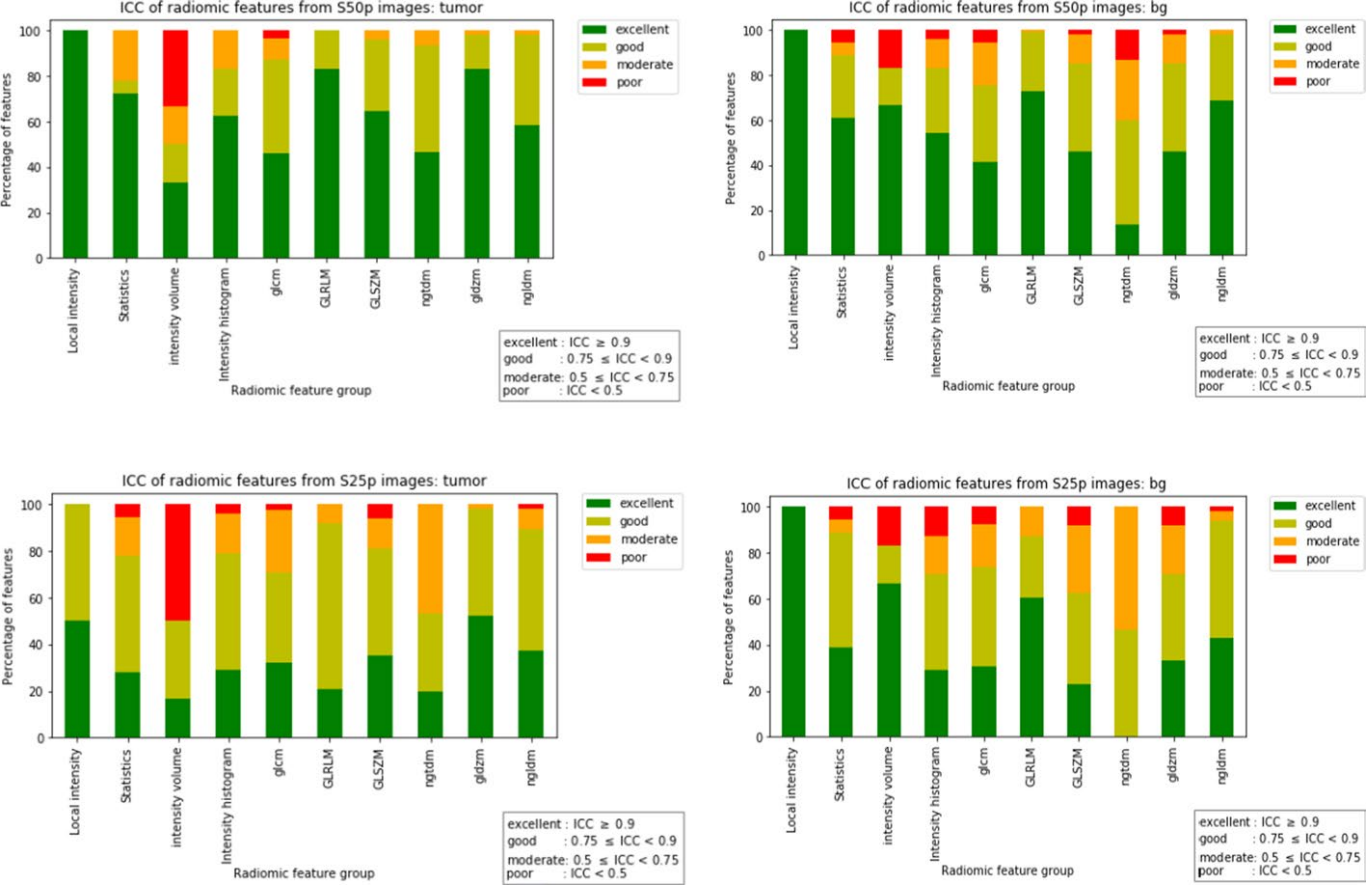
ICC of radiomic features per feature category for tumours (left) and BG (right) in S50p (top) and S25p (bottom) count-split images

For S50p, a total of 92% features are classified as having good (29%) or excellent (63%) ICC, while for S25p the total number decreased to 81%, with 49% as good and 32% as excellent ICC. The ICC results are summarised in Additional file 1: Table S2. While S50p had only 6% moderate ICC and 2% poor ICC features, S25p had 16% moderate ICC and 3% poor ICC features. These differences in ICC between S50p and S25p were statistically significant (*p* < 0.001). Per group, the percentage of features belonging to each of the ICC categories is shown in Fig. 1. Even though the local intensity family has the highest rate of features with good or excellent precision in both S50p and S25p images (100%), it should be noted that there are only two features in that feature family. GLRLM, GLDZM, and NGLDM families, which have relatively high numbers of features (see Additional file 1: Table S4), have the next highest percentage of features with good or excellent precision (100%, 98%, 98% in S50p and 92%, 98%, 90% in S25p images, respectively). The noise level affected the precision of features differently. For example, both GLRLM and GLDZM families have 83% features with excellent precision in S50p images. In S25p images, this percentage dropped but in different amounts for the two families (21% and 52%, respectively). However, the precision of 2D and 3D textural features were affected similarly. In GLRLM and GLDZM families, the ICC of 2D and 3D features varied between 81 and 88% for both feature groups in S50p images and decreased to 18 and 22% for GLRLM and to 50 and 53% for GLDZM in S25p images. The IVH family has the highest percentage of features with poor precision (17% for S50p and 50% for S25p). After IVH, NGTDM and GLCM families had the least percentages of features with excellent precision (47% and 46% in S50p and 20% and 32% in S25p, respectively). The ICC (mean and SD) for all the features can be found in Additional file 1: Table S5.

#### Analysis of precision (ICC) in BG

The precision of features in the BG tissues followed a similar pattern to that of tumour lesions, as shown in Fig. 1. For S50p, a total of 85% features were classified as having good (33%) or excellent (53%) ICC, while for S25p the total number decreased to 77% (40% as good and 38% as excellent ICC). While S50p had only 20% moderate ICC and 6% poor ICC features, S25p had 31% moderate ICC and 10% poor ICC features. These differences in ICC between S50p and S25p were statistically significant (*p* < 0.001).

### Analysis of noise-induced bias (SDM)

#### Analysis of noise-induced bias (SDM) in tumour lesions

**Fig. 2:**
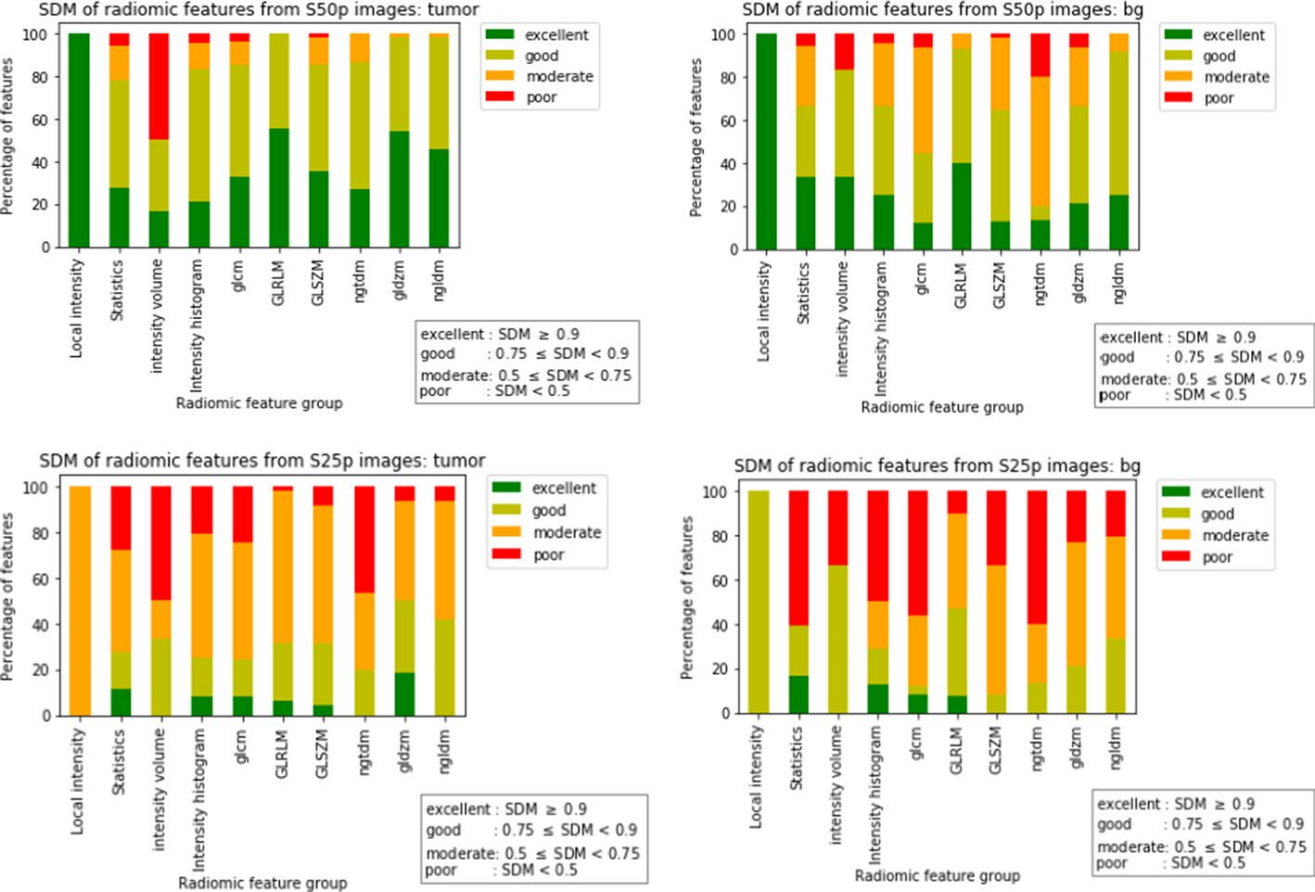
SDM of radiomic features per feature category for tumours (left) and BG (right) in S50p (top) and S25p (bottom) count-split images

For S50p, a total of 90% of features were classified as having a good (50%) or excellent (40%) SDM, while for S25p, the number decreased to 31%, with 24% as good and 7% as excellent SDM. The summary of SDM results is given in Additional file 1: Table S3. While S50p had only 9% moderate SDM and 3% poor SDM features, S25p had 54% moderate SDM and 15% poor SDM features. These differences in SDM were statistically significant (*p* < 0.001). Per group, the percentage of features in each SDM category is shown in Fig. 2. The SDM (mean and SD) for all the features can be found in Additional file 1: Table S5. The noise level affected the accuracy of features also differently. For example, GLRLM, GLDZM, and NGLDM families have the highest percentage of features with good or excellent accuracy in S50p images (100%, 98%, 98%, respectively) as can be seen in Additional file 1: Table S4. In S25p images, the highest noise level affected the accuracy of the features resulting in a drop in these percentages but different amounts (31%, 50%, and 42%, respectively). However, as in the previous case of precision, the accuracy of 2D and 3D textural features were affected similarly. In GLRLM and GLDZM families, the SDM of 2D and 3D features varied between 97 and 100% for both the feature groups in S50p images and decreased to 31% for GLRLM and to 50–53% for GLDZM in S25p images. Even though the local intensity and IVH families have the lowest percentage of features with good or excellent accuracy in S25p (0%) and S50p images (50%), it should be noted that only a few features belong to these feature families, as mentioned earlier in Sect. 3.1.1. GLCM and NGTDM families have the next lowest percentage of features with good or excellent accuracy in S25p images (24% and 20%, respectively), although these families perform better in low noise S50p images (85% and 87%, respectively).

#### Analysis of noise-induced bias (SDM) in BG

The accuracy of features in the BG tissues followed a similar pattern to that of tumour lesions, as shown in Fig. 2. For S50p, a total of 66% features were classified as having a good (44%) or excellent (22%) SDM, while for S25p, the total number decreased to 25% (20% as good and 5% as excellent SDM). While S50p had only 30% moderate SDM and 4% poor SDM features, S25p had 38% moderate SDM and 36% poor SDM features. These differences in SDM between S50p and S25p were statistically significant (p < 0.001).

**Fig. 3:**
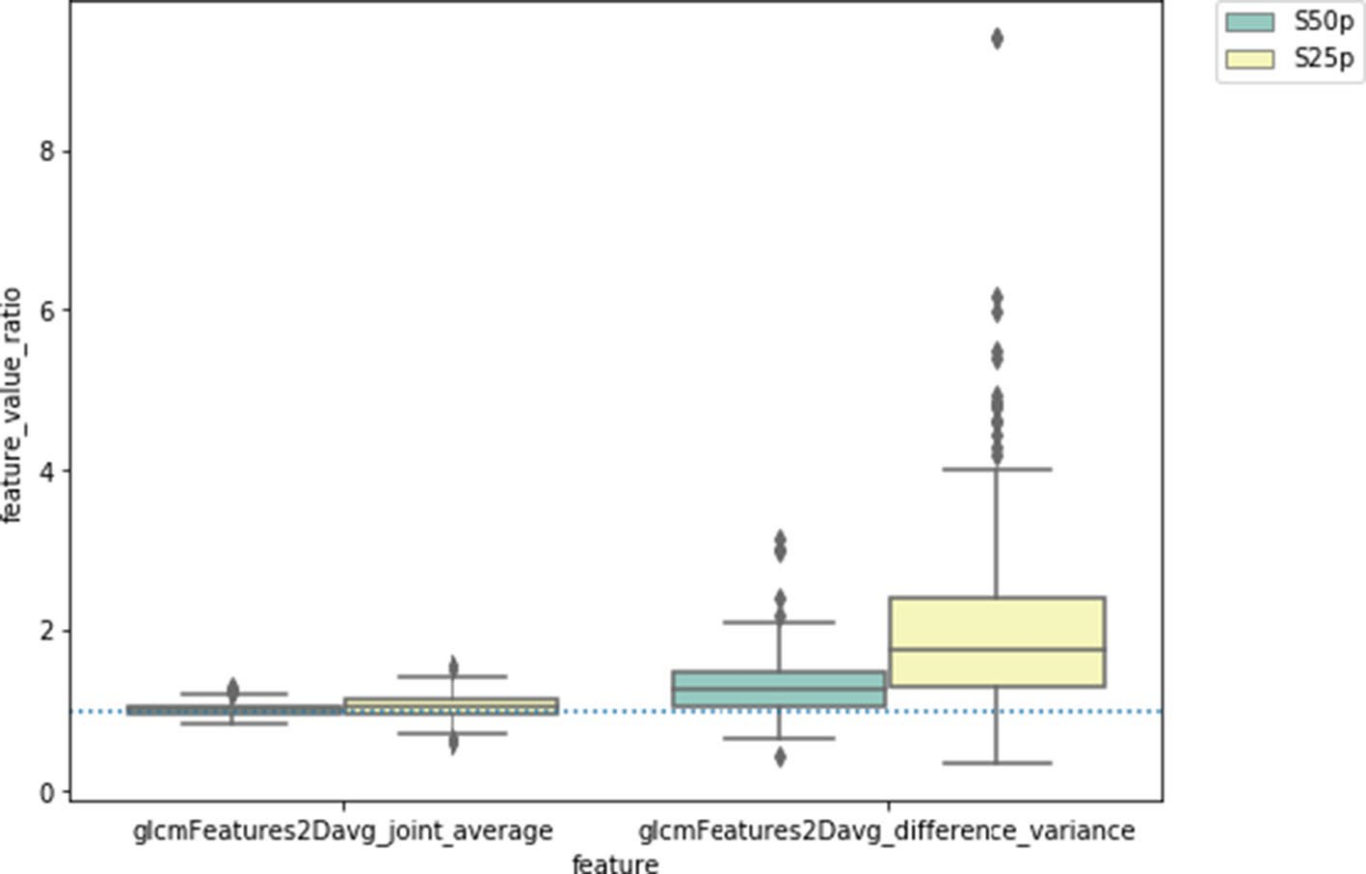
Boxplot showing radiomic features in S50p and S25p images normalised to full-count images. On the left is an example of a feature with low noise-induced variability and bias (ICC and SDM ≥ 0.9 for both S50p and S25p) illustrated by the narrow width of the box centred around the dotted line of unity, respectively. On the right is an example of a feature with high noise-induced variability and bias (ICC = 0.85 and 0.69, SDM = 0.85 and 0.49 for S50p and S25p, respectively) illustrated by the increase in the width of the box and the shift of the median from the line of unity in S25p, respectively

The number of features per feature group robust to noise in terms of both excellent ICC and SDM in S50p and S25p images are given in Table 1. Figure 3 illustrates the noise-induced variability and bias in two ^89^Zr-Immuno-PET derived radiomic features with the count-split features normalised to the full-count feature value.

## Discussion

To have clinical utility, a radiomic feature value should only depend on the tumour characteristics and not on noise. Otherwise, detecting treatment-induced variations based on a change in the radiomic feature value becomes unreliable. In the current study, the noise-induced errors due to variability and bias were assessed in the radiomic features derived from clinical ^89^Zr-Immuno-PET data. We have shown that noise causes variability between features in count statistically independent images and that there is a bias in the radiomic features in images with low SNR. Many radiomic features capture mainly noise rather than spatial arrangements. For, example, first-order statistics and intensity volume histograms only consider voxel value distributions without considering their locations. In other words, many features are mainly driven, or to a large extent affected by, noise in low count PET data.

A total of 148 features (33%) have shown robustness to noise with good or excellent ICC and SDM in both S50p and S25p images (see Additional file 1: Table S4). Two-dimensional and three-dimensional textural features were found to have similar noise-induced bias and variability. The ICC and SDM for a subset of the robust 148 features along with the 95% CI are shown in Additional file 1: Fig. S3 and S4. The 196 robust features (43%) that have excellent ICC and SDM for S50p images (listed in Additional file 1: Table S6) are a good starting point and have the potential to be used in the analysis of the clinical value of ^89^Zr-Immuno-PET derived radiomics. Moreover, only 33 features (7%) are robust to noise in S25p images. These can be considered for further clinical analysis of ^89^Zr-Immuno-PET radiomics in cases where the injected activity needs to be very low (e.g. non-oncological cases). These features can also be investigated for ^89^Zr-Immuno-PET with lower counts, e.g. when a later uptake time is of interest because of a longer biological half-life. Yet, in our paper, we focused primarily on S50p data because the photon counts, and therefore, the image quality in ^89^Zr-Immuno-PET scan on day 5 – 7 for oncological applications with an injected activity of 37 MBq is at least comparable to S50p images used in our study. In the Netherlands, the code of practice is that most ^89^Zr-Immuno-PET are conducted using a fixed activity level of 37 MBq (for radiation dose vs image quality reasons). Considering an uptake interval of 5 to 7 days for most antibodies, the decay would correspond to an activity of approximately 8 to 13 MBq at scan start. As indicated in Additional file 1: Table S1, the activity at scan start in our data is about 15 to 20 MBq. When using a count split of 50% (S50p), the count-reduction would correspond to an activity of about 7 to 10 MBq at scan start. Therefore, we can expect that the S50p data are representative for the counts and thus image quality observed in routine practice. Details of S25p data are, however, provided in the supplemental data (Additional file 1: Table S6).

The presence of noise-induced variability and bias in low-count PET images, as in ^89^Zr-Immuno-PET scans, introduces several challenges for using radiomic features. This intrinsic noise stresses the necessity to harmonise PET protocols, especially in the context of multicentre studies that involve different scanners and vendors, all with different noise sensitivity. While multicentre harmonisation of ^89^Zr-PET/CT SUV performance has been shown to be feasible [25], a more detailed study might be necessary to analyse the feasibility of multicentre harmonisation to optimise ^89^Zr-PET/CT radiomics performance. For example, to estimate the measurement error more accurately, phantoms can be used to study the noise dependency of PET radiomic features in more detail. One of the limitations of our current study is the small dataset consisting of only nine patients with mainly small tumours. Although a larger dataset is indeed required for developing an accurate clinical (diagnostic or predictive) model, we have tried to show noise sensitivity of radiomics independent of other characteristics and as such our conclusion that radiomics have noise-induced variability and bias remains the same. Previous studies have shown that measurement error depend on tumour characteristics such as type, shape, volume and tracer uptake and distribution characteristics [9, 12]. For example, radiomics extracted from large tumours and tumours with high uptake have been found to have better repeatability than those from small tumours and tumours with low uptake. Since our dataset contained only a small variety of tumours, the radiomic feature performance has to be further validated for different clinical applications. However, as we have primarily smaller and lower uptake lesions, we expect that for larger tumours and tracers with higher uptake more features would be classified as robust. Our study, therefore, provides a conservative (safe) assessment of robust features to be further clinically evaluated in ^89^Zr-Immuno-PET studies. Phantom experiments, in combination with ^89^Zr-Immuno-PET clinical studies, can be used to identify those radiomic features that are more robust to noise, patient, and tumour specific factors and to explore different techniques and scan protocols that provide robust radiomics.

While the present study has analysed the impact of image noise on the bias and variability of the radiomic features using count-split images, feature stability under the influence of other factors such as segmentation method or voxel size should be further investigated for ^89^Zr-Immuno PET because the stability of a feature in poor noise conditions does not generalise to different settings affecting the radiomic features. The low SNR in ^89^Zr-Immuno PET can especially make (semi-)automatic segmentations difficult to achieve. Manual delineations can also be expected to be more challenging and as a result, the extracted radiomic features less reproducible [14]. Administered activity and scan durations should therefore be chosen such that a minimal image quality is guaranteed. However, the features identified as robust to noise with minimal noise-induced variability and bias in this study provides a good starting point for these future analyses for the same reason: the accuracy and precision of these features are not affected by these confounding factors. This method of using ICC and SDM to select features robust to noise is also generally applicable to images from different scanners and tracers. ICC provides insight in precision, while SDM is a metric for bias and as such these are useful metrics to assess radiomics performance regardless of scanner or tracer being used.

Although many radiomic features have been shown to be highly correlated among themselves [12], feature correlation and subsequent feature selection have not been included in our analysis because our aim was to study the effects of noise on the performance of all individual radiomic features instead of only on a subset of features. Yet, feature selection based on, for example, correlations becomes important for the development of a diagnostic or clinical prediction model to avoid redundancy in the feature space, to reduce the number of features for model development, and enhancing the robustness of the model.

## Conclusion

Several radiomic features derived from low SNR ^89^Zr-Immuno-PET images have shown both noise-induced variability and bias, which should be accounted for before they can be used in predictive analysis to get meaningful results that are not noise-dependent. However, we were able to identify 196 features (43%) robust features that present a minimal noise-induced bias and acceptable ICCs across the 50% count-split levels. Therefore, these features seem to be minimally affected by noise and are particularly suited for radiomics analysis of PET studies under poor signal-to-noise conditions, such as ^89^Zr-Immuno-PET studies.

## Supplementary Information

**Additional file 1**. Supplementary Figures and Tables.

## Abbreviations

CI: Confidence Interval
EARL: European Association of Nuclear Medicine Research Ltd
FBW: Fixed bin width
FBN: Fixed bin number
IBSI: Imaging Biomarker Standardisation Initiative
ICC: Intraclass correlation coefficient
mAb: Monoclonal antibody
MATV: Metabolically active tumour volume
PET: Positron emission tomography
RC: Repeatability coefficient
SD: Standard deviation
SNR: Signal-to-noise ratio
SUV: Standardised uptake value
VOI: Volume of interest
Zr: Zirconium

## Declarations

### Availability of data and materials

All images analysed during this study are included in published articles [15–17]. The radiomic data used during the current study can be calculated and analysed as mentioned in the manuscript.
