# Supplementary material for: Noise sensitivity of 89Zr-Immuno-PET radiomics based on count-reduced clinical images
Source: EJNMMI Phys. 2022 Mar 3;9:16. doi: 10.1186/s40658-022-00444-4 (PMC8894530; doi:10.1186/s40658-022-00444-4)
Supplement: Supplementary file 1 — Additional file 1. Supplementary Figures and Tables. [file 40658_2022_444_MOESM1_ESM.docx]

**Supplement**


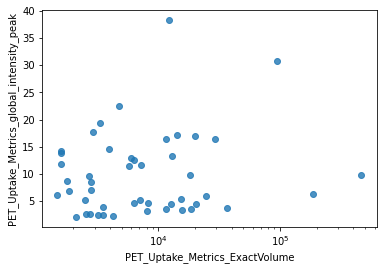


Fig S1. Scatterplot of SUVpeak (vertical axis) and volume in log scale (horizontal axis) of all the tumours

Table S1. Demographic and clinical data of all patients

| Patient | Gender | Weight (kg) | Height (cm) | Anti-mAb | # Tumours | Activity at scan start (Mbq) |
| --- | --- | --- | --- | --- | --- | --- |
| P01 | M | 98 | 196 | CD20 | 0 | 20.28 |
| P02 | F | 82 | 165 | CD20 | 6 | 21.59 |
| P03 | F | 69 | 172 | CD20 | 1 | 20.15 |
| P04 | M | 92 | 182 | CD20 | 22 | 20.44 |
| P05 | M | 73 | 177 | CD20 | 2 | 19.74 |
| P06 | F | 55 | 169 | CD44 | 0 | 15.59 |
| P07* | M | 72 | 176 | EGFR | 0 | 9.99 |
| P08 | M | 80 | 178 | CD44 | 0 | 15.38 |
| P09 | M | 90 | 170 | CD44 | 0 | 15.44 |
| P10 | M | 83 | 172 | CD44 | 0 | 15.62 |
| P11 | F | 62 | 176 | CD44 | 0 | 15.47 |
| P12 | M | 95 | 192 | CD44 | 2 | 15.24 |
| P13 | M | 56 | 173 | CD44 | 8 | 16.29 |
| P14 | M | 71 | 190 | CD44 | 3 | 15.72 |
| P15 | F | 65 | 167 | CD44 | 0 | 15.76 |
| P16 | M | 87 | 179 | CD44 | 1 | 15.64 |
| P17 | M | 90 | 182 | CD44 | 2 | 13.37 |
| P18* | M | 79 | 175 | EGFR | 0 | 9.92 |
| P19* | M | 82 | 182 | EGFR | 2 | 10.15 |
| P20* | M | 88 | 180 | CD20 | 1 | NA |

(*Patients excluded from the study because of unavailable data or because of low activity at scan start)


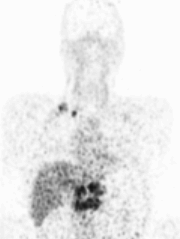

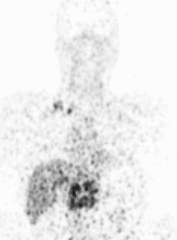

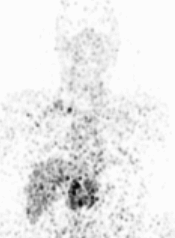


Fig. S2. A single coronal slice of an ^89^Zr-Immuno-PET patient scan reconstructed with 100% (original), 50% (S50p) and 25% (S25p) counts (from left to right)

Table S2. Number of features belonging to each ICC category in S50p and S25p images for tumour lesions and BG tissues

| ICC Category | S50p | | S25p | |
| --- | --- | --- | --- | --- |
|  | Tumour | BG | Tumour | BG |
| Excellent | 302 | 260 | 159 | 188 |
| Good | 135 | 148 | 229 | 183 |
| Moderate | 33 | 51 | 76 | 79 |
| Poor | 7 | 17 | 13 | 28 |

Table S3. Number of features belonging to each SDM category in S50p and S25p images for tumour lesions and BG tissues

| SDM Category | S50p | | S25p | |
| --- | --- | --- | --- | --- |
|  | Tumour | BG | Tumour | BG |
| Excellent | 184 | 102 | 33 | 25 |
| Good | 227 | 119 | 108 | 90 |
| Moderate | 32 | 134 | 245 | 175 |
| Poor | 12 | 20 | 69 | 165 |

Table S4. Radiomic feature groups with the number of good and/or excellent features in S50p and/or S25p images

| Radiomic feature group | # Total radiomic features | # Good or excellent ICC features in S50p and S25p | # Good or excellent SDM features in S50p and S25p | # Good or excellent ICC and SDM features in S50p and S25p | # Good or excellent ICC and SDM features in S50p | # Good or excellent ICC and SDM features in S25p |
| --- | --- | --- | --- | --- | --- | --- |
| Local intensity | 2 | 2 | 0 | 0 | 2 | 0 |
| Statistics | 18 | 14 | 5 | 5 | 14 | 5 |
| Intensity histogram | 24 | 19 | 6 | 6 | 20 | 6 |
| Intensity volume histogram | 6 | 3 | 2 | 2 | 3 | 2 |
| Grey level co-occurrence (GLCM) | 150 | 107 | 41 | 41 | 132 | 41 |
| Grey level run length (GLRLM) | 96 | 90 | 30 | 30 | 96 | 30 |
| Grey level size zone (GLSZM) | 48 | 39 | 15 | 15 | 40 | 15 |
| Grey level distance zone (GLDZM) | 48 | 47 | 25 | 25 | 47 | 25 |
| Neighbourhood grey tone difference (NGTDM) | 15 | 9 | 3 | 3 | 13 | 3 |
| Neighbouring grey level dependence (NGLDM) | 51 | 43 | 21 | 21 | 47 | 21 |
| Total | 458 | 373 | 148 | 148 | 414 | 148 |

Table S5. List of radiomic features calculated as per IBSI guidelines with corresponding mean ICC and SDM for S50p and S25p images

| **feature_group** | **feature_name** | **S50p_ICC** | | **S25p_ICC** | | **S50p_SDM** | | **S25p_SDM** | |
| --- | --- | --- | --- | --- | --- | --- | --- | --- | --- |
|  |  | **mean** | **SD** | **mean** | **SD** | **mean** | **SD** | **mean** | **SD** |
| Local intensity | local intensity peak | 0.95 | 0.02 | 0.88 | 0.04 | 0.91 | 0.01 | 0.73 | 0.08 |
| Local intensity | global intensity peak | 0.97 | 0.01 | 0.92 | 0.03 | 0.93 | 0.01 | 0.71 | 0.05 |
| Statistics | mean | 0.96 | 0.02 | 0.93 | 0.03 | 0.96 | 0.01 | 0.9 | 0.03 |
| Statistics | variance | 0.89 | 0.06 | 0.78 | 0.13 | 0.86 | 0.04 | 0.51 | 0.11 |
| Statistics | skewness | 0.61 | 0.08 | 0.53 | 0.03 | 0.56 | 0.03 | 0.27 | 0.05 |
| Statistics | kurtosis | 0.58 | 0.19 | 0.41 | 0.06 | 0.43 | 0.05 | 0.16 | 0.08 |
| Statistics | median | 0.97 | 0.02 | 0.92 | 0.03 | 0.96 | 0.01 | 0.91 | 0.03 |
| Statistics | minimum | 0.94 | 0.03 | 0.79 | 0.07 | 0.87 | 0.02 | 0.71 | 0.05 |
| Statistics | 10th percentile | 0.94 | 0.03 | 0.9 | 0.05 | 0.94 | 0.02 | 0.87 | 0.04 |
| Statistics | 90th percentile | 0.96 | 0.02 | 0.92 | 0.04 | 0.95 | 0.02 | 0.82 | 0.04 |
| Statistics | maximum | 0.93 | 0.02 | 0.89 | 0.04 | 0.85 | 0.01 | 0.55 | 0.06 |
| Statistics | Interquartile range | 0.93 | 0.04 | 0.85 | 0.06 | 0.89 | 0.04 | 0.64 | 0.08 |
| Statistics | range | 0.92 | 0.03 | 0.88 | 0.05 | 0.82 | 0.02 | 0.47 | 0.06 |
| Statistics | Mean absolut deviation | 0.92 | 0.04 | 0.87 | 0.05 | 0.88 | 0.04 | 0.61 | 0.07 |
| Statistics | Robust mean absolute deviation | 0.94 | 0.04 | 0.86 | 0.06 | 0.89 | 0.03 | 0.63 | 0.09 |
| Statistics | Median absolute deviation | 0.93 | 0.04 | 0.87 | 0.05 | 0.89 | 0.03 | 0.62 | 0.07 |
| Statistics | Coefficient of variation | 0.65 | 0.05 | 0.66 | 0.06 | 0.51 | 0.13 | 0.15 | 0.02 |
| Statistics | Quartile coefficient | 0.58 | 0.09 | 0.6 | 0.05 | 0.52 | 0.12 | 0.2 | 0.03 |
| Statistics | Energy | 0.99 | 0 | 0.99 | 0 | 0.79 | 0.05 | 0.69 | 0.07 |
| Statistics | Root mean | 0.96 | 0.02 | 0.92 | 0.03 | 0.96 | 0.01 | 0.88 | 0.03 |
| intensity volume | volume at int fraction 10 | 0 | 0 | 0.38 | 0.09 | 0.46 | 0.19 | 0.48 | 0.11 |
| intensity volume | volume at int fraction 90 | 0.59 | 0.04 | 0.37 | 0.07 | 0.47 | 0.03 | 0.46 | 0.05 |
| intensity volume | int at vol fraction 10 | 0.93 | 0.03 | 0.92 | 0.03 | 0.94 | 0.02 | 0.82 | 0.04 |
| intensity volume | int at vol fraction 90 | 0.89 | 0.04 | 0.87 | 0.06 | 0.87 | 0.02 | 0.79 | 0.04 |
| intensity volume | difference vol at int fraction | 0 | 0 | 0.36 | 0.1 | 0.45 | 0.19 | 0.49 | 0.12 |
| intensity volume | difference int at volume fraction | 0.91 | 0.04 | 0.87 | 0.05 | 0.88 | 0.04 | 0.6 | 0.06 |
| Intensity histogram | mean | 0.96 | 0.02 | 0.93 | 0.03 | 0.96 | 0.01 | 0.9 | 0.03 |
| Intensity histogram | variance | 0.89 | 0.06 | 0.78 | 0.13 | 0.86 | 0.04 | 0.51 | 0.11 |
| Intensity histogram | skewness | 0.63 | 0.08 | 0.53 | 0.03 | 0.54 | 0.02 | 0.27 | 0.05 |
| Intensity histogram | kurtosis | 0.58 | 0.19 | 0.41 | 0.06 | 0.44 | 0.05 | 0.16 | 0.08 |
| Intensity histogram | median | 0.96 | 0.02 | 0.92 | 0.03 | 0.96 | 0.01 | 0.91 | 0.03 |
| Intensity histogram | minimum | 0.93 | 0.04 | 0.8 | 0.07 | 0.86 | 0.02 | 0.71 | 0.04 |
| Intensity histogram | 10th percentile | 0.94 | 0.03 | 0.9 | 0.05 | 0.93 | 0.02 | 0.87 | 0.04 |
| Intensity histogram | 90th percentile | 0.95 | 0.02 | 0.92 | 0.03 | 0.95 | 0.02 | 0.82 | 0.04 |
| Intensity histogram | maximum | 0.94 | 0.02 | 0.89 | 0.04 | 0.85 | 0.01 | 0.55 | 0.06 |
| Intensity histogram | mode | 0.95 | 0.03 | 0.85 | 0.06 | 0.92 | 0.03 | 0.82 | 0.06 |
| Intensity histogram | Interquartile range | 0.93 | 0.04 | 0.85 | 0.06 | 0.87 | 0.03 | 0.63 | 0.09 |
| Intensity histogram | range | 0.92 | 0.02 | 0.88 | 0.05 | 0.82 | 0.02 | 0.47 | 0.06 |
| Intensity histogram | Mean absolut deviation | 0.92 | 0.04 | 0.87 | 0.05 | 0.88 | 0.03 | 0.61 | 0.07 |
| Intensity histogram | Robust mean absolute deviation | 0.93 | 0.04 | 0.86 | 0.06 | 0.89 | 0.03 | 0.63 | 0.08 |
| Intensity histogram | Median absolut deviation | 0.93 | 0.04 | 0.87 | 0.05 | 0.89 | 0.03 | 0.62 | 0.07 |
| Intensity histogram | Coefficient of variation | 0.66 | 0.06 | 0.67 | 0.06 | 0.52 | 0.13 | 0.16 | 0.02 |
| Intensity histogram | Quartile coefficient | 0.58 | 0.09 | 0.58 | 0.05 | 0.5 | 0.11 | 0.21 | 0.03 |
| Intensity histogram | Entropy | 0.93 | 0.02 | 0.92 | 0.02 | 0.88 | 0.05 | 0.69 | 0.04 |
| Intensity histogram | Uniformity | 0.88 | 0.01 | 0.84 | 0.01 | 0.87 | 0.06 | 0.69 | 0.05 |
| Intensity histogram | Energy | 0.99 | 0 | 0.99 | 0 | 0.79 | 0.05 | 0.7 | 0.07 |
| Intensity histogram | Maximum histogram gradient | 0.98 | 0.01 | 0.96 | 0.01 | 0.86 | 0.01 | 0.77 | 0.03 |
| Intensity histogram | Maximum histogram gradient grey level | 0.8 | 0.08 | 0.79 | 0.06 | 0.77 | 0.07 | 0.72 | 0.08 |
| Intensity histogram | Minimum histogram gradient | 0.86 | 0.02 | 0.93 | 0.03 | 0.85 | 0.05 | 0.73 | 0.03 |
| Intensity histogram | Minimum histogram gradient grey level | 0.84 | 0.11 | 0.72 | 0.08 | 0.8 | 0.06 | 0.73 | 0.05 |
| glcmFeatures2Davg | joint maximum | 0.9 | 0.01 | 0.82 | 0.02 | 0.9 | 0.02 | 0.72 | 0.06 |
| glcmFeatures2Davg | joint average | 0.96 | 0.02 | 0.93 | 0.03 | 0.96 | 0.01 | 0.9 | 0.02 |
| glcmFeatures2Davg | joint variance | 0.84 | 0.08 | 0.74 | 0.13 | 0.77 | 0.05 | 0.37 | 0.09 |
| glcmFeatures2Davg | joint entropy | 0.96 | 0.01 | 0.97 | 0 | 0.9 | 0.02 | 0.74 | 0.02 |
| glcmFeatures2Davg | difference average | 0.92 | 0.03 | 0.84 | 0.05 | 0.92 | 0.01 | 0.7 | 0.05 |
| glcmFeatures2Davg | difference variance | 0.85 | 0.06 | 0.69 | 0.16 | 0.85 | 0.04 | 0.49 | 0.12 |
| glcmFeatures2Davg | difference entropy | 0.94 | 0.01 | 0.93 | 0.01 | 0.88 | 0.03 | 0.66 | 0.02 |
| glcmFeatures2Davg | sum average | 0.96 | 0.02 | 0.93 | 0.03 | 0.96 | 0.01 | 0.9 | 0.02 |
| glcmFeatures2Davg | sum variance | 0.84 | 0.08 | 0.75 | 0.12 | 0.75 | 0.05 | 0.34 | 0.08 |
| glcmFeatures2Davg | sum entropy | 0.95 | 0.01 | 0.96 | 0.01 | 0.88 | 0.04 | 0.7 | 0.02 |
| glcmFeatures2Davg | angular second moment | 0.87 | 0.01 | 0.85 | 0.01 | 0.85 | 0 | 0.69 | 0.04 |
| glcmFeatures2Davg | contrast | 0.85 | 0.05 | 0.68 | 0.13 | 0.87 | 0.04 | 0.55 | 0.14 |
| glcmFeatures2Davg | dissimilarity | 0.92 | 0.03 | 0.84 | 0.05 | 0.92 | 0.01 | 0.7 | 0.05 |
| glcmFeatures2Davg | inverse difference | 0.92 | 0.01 | 0.92 | 0.01 | 0.91 | 0.03 | 0.75 | 0.01 |
| glcmFeatures2Davg | inverse difference normalised | 0.82 | 0.02 | 0.84 | 0.02 | 0.87 | 0.01 | 0.78 | 0.04 |
| glcmFeatures2Davg | inverse difference moment | 0.91 | 0.01 | 0.92 | 0.01 | 0.9 | 0.03 | 0.74 | 0.02 |
| glcmFeatures2Davg | inverse difference moment normalised | 0.78 | 0.02 | 0.82 | 0.02 | 0.86 | 0.03 | 0.79 | 0.04 |
| glcmFeatures2Davg | inverse variance | 0.88 | 0.02 | 0.93 | 0.02 | 0.87 | 0.05 | 0.72 | 0.02 |
| glcmFeatures2Davg | correlation | 0.87 | 0.02 | 0.87 | 0.03 | 0.83 | 0.05 | 0.68 | 0.05 |
| glcmFeatures2Davg | autocorrelation | 0.95 | 0.03 | 0.88 | 0.07 | 0.95 | 0.02 | 0.85 | 0.06 |
| glcmFeatures2Davg | cluster tendency | 0.84 | 0.08 | 0.75 | 0.12 | 0.75 | 0.05 | 0.34 | 0.08 |
| glcmFeatures2Davg | cluster shade | 0.61 | 0.29 | 0.57 | 0.35 | 0.48 | 0.1 | 0.12 | 0.07 |
| glcmFeatures2Davg | cluster prominence | 0.7 | 0.19 | 0.59 | 0.24 | 0.58 | 0.12 | 0.18 | 0.1 |
| glcmFeatures2Davg | first measure of information correlation | 0.8 | 0.07 | 0.88 | 0.04 | 0.8 | 0.09 | 0.66 | 0.04 |
| glcmFeatures2Davg | second measure of information correlation | 0.82 | 0.02 | 0.82 | 0.02 | 0.77 | 0.01 | 0.61 | 0.06 |
| glcmFeatures2DDmrg | joint maximum | 0.91 | 0.01 | 0.7 | 0.01 | 0.9 | 0.01 | 0.7 | 0.09 |
| glcmFeatures2DDmrg | joint average | 0.96 | 0.02 | 0.92 | 0.03 | 0.96 | 0.01 | 0.9 | 0.03 |
| glcmFeatures2DDmrg | joint variance | 0.88 | 0.06 | 0.77 | 0.14 | 0.85 | 0.04 | 0.5 | 0.11 |
| glcmFeatures2DDmrg | joint entropy | 0.94 | 0.01 | 0.94 | 0.01 | 0.88 | 0.04 | 0.7 | 0.03 |
| glcmFeatures2DDmrg | difference average | 0.9 | 0.04 | 0.83 | 0.06 | 0.91 | 0.02 | 0.71 | 0.06 |
| glcmFeatures2DDmrg | difference variance | 0.81 | 0.07 | 0.67 | 0.17 | 0.82 | 0.05 | 0.48 | 0.13 |
| glcmFeatures2DDmrg | difference entropy | 0.93 | 0.02 | 0.91 | 0.02 | 0.9 | 0.03 | 0.7 | 0.03 |
| glcmFeatures2DDmrg | sum average | 0.96 | 0.02 | 0.92 | 0.03 | 0.96 | 0.01 | 0.9 | 0.03 |
| glcmFeatures2DDmrg | sum variance | 0.89 | 0.06 | 0.77 | 0.14 | 0.85 | 0.04 | 0.49 | 0.11 |
| glcmFeatures2DDmrg | sum entropy | 0.92 | 0.02 | 0.92 | 0.02 | 0.87 | 0.05 | 0.68 | 0.04 |
| glcmFeatures2DDmrg | angular second moment | 0.88 | 0.01 | 0.76 | 0.01 | 0.88 | 0.04 | 0.69 | 0.07 |
| glcmFeatures2DDmrg | contrast | 0.82 | 0.07 | 0.67 | 0.15 | 0.85 | 0.04 | 0.56 | 0.14 |
| glcmFeatures2DDmrg | dissimilarity | 0.9 | 0.04 | 0.83 | 0.06 | 0.91 | 0.02 | 0.71 | 0.06 |
| glcmFeatures2DDmrg | inverse difference | 0.93 | 0.01 | 0.92 | 0.01 | 0.91 | 0.03 | 0.75 | 0.02 |
| glcmFeatures2DDmrg | inverse difference normalised | 0.89 | 0.02 | 0.83 | 0.02 | 0.89 | 0.01 | 0.79 | 0.05 |
| glcmFeatures2DDmrg | inverse difference moment | 0.93 | 0.01 | 0.92 | 0.01 | 0.9 | 0.04 | 0.75 | 0.02 |
| glcmFeatures2DDmrg | inverse difference moment normalised | 0.89 | 0.03 | 0.81 | 0.02 | 0.9 | 0.02 | 0.79 | 0.06 |
| glcmFeatures2DDmrg | inverse variance | 0.89 | 0.02 | 0.93 | 0.01 | 0.87 | 0.04 | 0.73 | 0.02 |
| glcmFeatures2DDmrg | correlation | 0.8 | 0.05 | 0.67 | 0.04 | 0.82 | 0.03 | 0.64 | 0.08 |
| glcmFeatures2DDmrg | autocorrelation | 0.94 | 0.04 | 0.87 | 0.08 | 0.95 | 0.02 | 0.84 | 0.08 |
| glcmFeatures2DDmrg | cluster tendency | 0.89 | 0.06 | 0.77 | 0.14 | 0.85 | 0.04 | 0.49 | 0.11 |
| glcmFeatures2DDmrg | cluster shade | 0.49 | 0.29 | 0.46 | 0.24 | 0.5 | 0.14 | 0.14 | 0.11 |
| glcmFeatures2DDmrg | cluster prominence | 0.71 | 0.13 | 0.56 | 0.23 | 0.67 | 0.15 | 0.27 | 0.17 |
| glcmFeatures2DDmrg | first measure of information correlation | 0.83 | 0.08 | 0.8 | 0.06 | 0.79 | 0.07 | 0.58 | 0.09 |
| glcmFeatures2DDmrg | second measure of information correlation | 0.72 | 0.03 | 0.7 | 0.03 | 0.72 | 0.11 | 0.55 | 0.04 |
| glcmFeatures2Dmrg | joint maximum | 0.89 | 0.01 | 0.8 | 0.02 | 0.9 | 0.02 | 0.71 | 0.06 |
| glcmFeatures2Dmrg | joint average | 0.96 | 0.02 | 0.93 | 0.03 | 0.96 | 0.01 | 0.9 | 0.02 |
| glcmFeatures2Dmrg | joint variance | 0.84 | 0.08 | 0.74 | 0.13 | 0.77 | 0.05 | 0.37 | 0.09 |
| glcmFeatures2Dmrg | joint entropy | 0.93 | 0.01 | 0.95 | 0.01 | 0.87 | 0.03 | 0.69 | 0.02 |
| glcmFeatures2Dmrg | difference average | 0.92 | 0.03 | 0.84 | 0.05 | 0.92 | 0.01 | 0.69 | 0.05 |
| glcmFeatures2Dmrg | difference variance | 0.86 | 0.06 | 0.67 | 0.15 | 0.85 | 0.04 | 0.47 | 0.13 |
| glcmFeatures2Dmrg | difference entropy | 0.93 | 0.01 | 0.92 | 0.02 | 0.89 | 0.03 | 0.69 | 0.02 |
| glcmFeatures2Dmrg | sum average | 0.96 | 0.02 | 0.93 | 0.03 | 0.96 | 0.01 | 0.9 | 0.02 |
| glcmFeatures2Dmrg | sum variance | 0.84 | 0.08 | 0.75 | 0.12 | 0.75 | 0.05 | 0.34 | 0.08 |
| glcmFeatures2Dmrg | sum entropy | 0.91 | 0.02 | 0.93 | 0.01 | 0.86 | 0.04 | 0.66 | 0.02 |
| glcmFeatures2Dmrg | angular second moment | 0.86 | 0.01 | 0.82 | 0.02 | 0.84 | 0 | 0.68 | 0.04 |
| glcmFeatures2Dmrg | contrast | 0.85 | 0.05 | 0.68 | 0.13 | 0.87 | 0.04 | 0.55 | 0.14 |
| glcmFeatures2Dmrg | dissimilarity | 0.92 | 0.03 | 0.84 | 0.05 | 0.92 | 0.01 | 0.69 | 0.05 |
| glcmFeatures2Dmrg | inverse difference | 0.92 | 0.01 | 0.92 | 0.01 | 0.91 | 0.03 | 0.74 | 0.01 |
| glcmFeatures2Dmrg | inverse difference normalised | 0.82 | 0.02 | 0.84 | 0.02 | 0.87 | 0.01 | 0.78 | 0.04 |
| glcmFeatures2Dmrg | inverse difference moment | 0.91 | 0.01 | 0.92 | 0.01 | 0.9 | 0.03 | 0.74 | 0.02 |
| glcmFeatures2Dmrg | inverse difference moment normalised | 0.78 | 0.02 | 0.82 | 0.02 | 0.86 | 0.03 | 0.79 | 0.04 |
| glcmFeatures2Dmrg | inverse variance | 0.88 | 0.02 | 0.93 | 0.02 | 0.87 | 0.05 | 0.72 | 0.02 |
| glcmFeatures2Dmrg | correlation | 0.87 | 0.03 | 0.86 | 0.03 | 0.82 | 0.05 | 0.68 | 0.05 |
| glcmFeatures2Dmrg | autocorrelation | 0.95 | 0.03 | 0.88 | 0.07 | 0.95 | 0.02 | 0.85 | 0.06 |
| glcmFeatures2Dmrg | cluster tendency | 0.84 | 0.08 | 0.75 | 0.12 | 0.75 | 0.05 | 0.34 | 0.08 |
| glcmFeatures2Dmrg | cluster shade | 0.61 | 0.29 | 0.56 | 0.35 | 0.48 | 0.1 | 0.12 | 0.07 |
| glcmFeatures2Dmrg | cluster prominence | 0.7 | 0.19 | 0.59 | 0.24 | 0.58 | 0.12 | 0.18 | 0.1 |
| glcmFeatures2Dmrg | first measure of information correlation | 0.41 | 0.23 | 0.64 | 0.13 | 0.61 | 0.18 | 0.45 | 0.07 |
| glcmFeatures2Dmrg | second measure of information correlation | 0.75 | 0.04 | 0.85 | 0.02 | 0.78 | 0.04 | 0.62 | 0.04 |
| glcmFeatures2Dvmrg | joint maximum | 0.9 | 0.01 | 0.67 | 0.02 | 0.89 | 0.01 | 0.69 | 0.1 |
| glcmFeatures2Dvmrg | joint average | 0.96 | 0.02 | 0.92 | 0.03 | 0.96 | 0.01 | 0.9 | 0.03 |
| glcmFeatures2Dvmrg | joint variance | 0.88 | 0.06 | 0.77 | 0.14 | 0.85 | 0.04 | 0.5 | 0.11 |
| glcmFeatures2Dvmrg | joint entropy | 0.94 | 0.01 | 0.94 | 0.01 | 0.89 | 0.04 | 0.71 | 0.03 |
| glcmFeatures2Dvmrg | difference average | 0.9 | 0.03 | 0.84 | 0.06 | 0.91 | 0.02 | 0.71 | 0.06 |
| glcmFeatures2Dvmrg | difference variance | 0.82 | 0.08 | 0.66 | 0.17 | 0.82 | 0.05 | 0.48 | 0.13 |
| glcmFeatures2Dvmrg | difference entropy | 0.93 | 0.02 | 0.91 | 0.02 | 0.9 | 0.03 | 0.71 | 0.03 |
| glcmFeatures2Dvmrg | sum average | 0.96 | 0.02 | 0.92 | 0.03 | 0.96 | 0.01 | 0.9 | 0.03 |
| glcmFeatures2Dvmrg | sum variance | 0.89 | 0.06 | 0.77 | 0.14 | 0.85 | 0.04 | 0.49 | 0.11 |
| glcmFeatures2Dvmrg | sum entropy | 0.92 | 0.02 | 0.91 | 0.02 | 0.88 | 0.05 | 0.68 | 0.04 |
| glcmFeatures2Dvmrg | angular second moment | 0.88 | 0.01 | 0.75 | 0 | 0.87 | 0.05 | 0.68 | 0.07 |
| glcmFeatures2Dvmrg | contrast | 0.82 | 0.07 | 0.67 | 0.15 | 0.85 | 0.04 | 0.56 | 0.14 |
| glcmFeatures2Dvmrg | dissimilarity | 0.9 | 0.03 | 0.84 | 0.06 | 0.91 | 0.02 | 0.71 | 0.06 |
| glcmFeatures2Dvmrg | inverse difference | 0.93 | 0.01 | 0.92 | 0.01 | 0.91 | 0.03 | 0.75 | 0.02 |
| glcmFeatures2Dvmrg | inverse difference normalised | 0.89 | 0.02 | 0.83 | 0.03 | 0.89 | 0.01 | 0.79 | 0.05 |
| glcmFeatures2Dvmrg | inverse difference moment | 0.93 | 0.01 | 0.92 | 0.01 | 0.9 | 0.04 | 0.75 | 0.02 |
| glcmFeatures2Dvmrg | inverse difference moment normalised | 0.89 | 0.03 | 0.81 | 0.03 | 0.9 | 0.02 | 0.79 | 0.06 |
| glcmFeatures2Dvmrg | inverse variance | 0.89 | 0.02 | 0.93 | 0.01 | 0.87 | 0.04 | 0.73 | 0.02 |
| glcmFeatures2Dvmrg | correlation | 0.81 | 0.05 | 0.67 | 0.04 | 0.81 | 0.03 | 0.63 | 0.08 |
| glcmFeatures2Dvmrg | autocorrelation | 0.94 | 0.04 | 0.87 | 0.08 | 0.95 | 0.02 | 0.84 | 0.08 |
| glcmFeatures2Dvmrg | cluster tendency | 0.89 | 0.06 | 0.77 | 0.14 | 0.85 | 0.04 | 0.49 | 0.11 |
| glcmFeatures2Dvmrg | cluster shade | 0.49 | 0.29 | 0.46 | 0.24 | 0.5 | 0.14 | 0.14 | 0.11 |
| glcmFeatures2Dvmrg | cluster prominence | 0.71 | 0.13 | 0.56 | 0.23 | 0.67 | 0.15 | 0.27 | 0.17 |
| glcmFeatures2Dvmrg | first measure of information correlation | 0.63 | 0.1 | 0.56 | 0.1 | 0.73 | 0.03 | 0.53 | 0.07 |
| glcmFeatures2Dvmrg | second measure of information correlation | 0.66 | 0.06 | 0.62 | 0.05 | 0.71 | 0.11 | 0.52 | 0.06 |
| glcmFeatures3Davg | joint maximum | 0.86 | 0.01 | 0.68 | 0.01 | 0.89 | 0.02 | 0.69 | 0.1 |
| glcmFeatures3Davg | joint average | 0.96 | 0.02 | 0.92 | 0.03 | 0.96 | 0.01 | 0.9 | 0.03 |
| glcmFeatures3Davg | joint variance | 0.88 | 0.07 | 0.76 | 0.14 | 0.85 | 0.04 | 0.49 | 0.11 |
| glcmFeatures3Davg | joint entropy | 0.94 | 0.01 | 0.94 | 0.01 | 0.88 | 0.03 | 0.7 | 0.03 |
| glcmFeatures3Davg | difference average | 0.91 | 0.04 | 0.84 | 0.06 | 0.91 | 0.02 | 0.71 | 0.06 |
| glcmFeatures3Davg | difference variance | 0.81 | 0.09 | 0.68 | 0.18 | 0.82 | 0.05 | 0.49 | 0.12 |
| glcmFeatures3Davg | difference entropy | 0.94 | 0.02 | 0.92 | 0.02 | 0.9 | 0.03 | 0.71 | 0.03 |
| glcmFeatures3Davg | sum average | 0.96 | 0.02 | 0.92 | 0.03 | 0.96 | 0.01 | 0.9 | 0.03 |
| glcmFeatures3Davg | sum variance | 0.88 | 0.07 | 0.76 | 0.14 | 0.84 | 0.04 | 0.48 | 0.11 |
| glcmFeatures3Davg | sum entropy | 0.92 | 0.02 | 0.92 | 0.02 | 0.87 | 0.05 | 0.68 | 0.04 |
| glcmFeatures3Davg | angular second moment | 0.85 | 0.01 | 0.74 | 0 | 0.87 | 0.05 | 0.68 | 0.08 |
| glcmFeatures3Davg | contrast | 0.83 | 0.08 | 0.68 | 0.16 | 0.85 | 0.04 | 0.56 | 0.14 |
| glcmFeatures3Davg | dissimilarity | 0.91 | 0.04 | 0.84 | 0.06 | 0.91 | 0.02 | 0.71 | 0.06 |
| glcmFeatures3Davg | inverse difference | 0.94 | 0.01 | 0.92 | 0.01 | 0.92 | 0.03 | 0.76 | 0.03 |
| glcmFeatures3Davg | inverse difference normalised | 0.92 | 0.01 | 0.85 | 0.02 | 0.9 | 0.01 | 0.81 | 0.07 |
| glcmFeatures3Davg | inverse difference moment | 0.94 | 0.01 | 0.92 | 0.01 | 0.91 | 0.03 | 0.75 | 0.03 |
| glcmFeatures3Davg | inverse difference moment normalised | 0.91 | 0.02 | 0.82 | 0.03 | 0.88 | 0.01 | 0.8 | 0.06 |
| glcmFeatures3Davg | inverse variance | 0.94 | 0.01 | 0.93 | 0.01 | 0.9 | 0.03 | 0.75 | 0.03 |
| glcmFeatures3Davg | correlation | 0.77 | 0.05 | 0.72 | 0.04 | 0.81 | 0.06 | 0.65 | 0.05 |
| glcmFeatures3Davg | autocorrelation | 0.94 | 0.04 | 0.87 | 0.08 | 0.95 | 0.02 | 0.84 | 0.08 |
| glcmFeatures3Davg | cluster tendency | 0.88 | 0.07 | 0.76 | 0.14 | 0.84 | 0.04 | 0.48 | 0.11 |
| glcmFeatures3Davg | cluster shade | 0.47 | 0.28 | 0.46 | 0.24 | 0.49 | 0.14 | 0.14 | 0.1 |
| glcmFeatures3Davg | cluster prominence | 0.7 | 0.14 | 0.56 | 0.23 | 0.66 | 0.16 | 0.26 | 0.17 |
| glcmFeatures3Davg | first measure of information correlation | 0.83 | 0.07 | 0.84 | 0.05 | 0.8 | 0.1 | 0.6 | 0.09 |
| glcmFeatures3Davg | second measure of information correlation | 0.71 | 0.04 | 0.75 | 0.03 | 0.71 | 0.13 | 0.55 | 0.03 |
| glcmFeatures3DWmrg | joint maximum | 0.86 | 0.01 | 0.63 | 0.02 | 0.89 | 0.01 | 0.68 | 0.11 |
| glcmFeatures3DWmrg | joint average | 0.96 | 0.02 | 0.92 | 0.03 | 0.96 | 0.01 | 0.9 | 0.03 |
| glcmFeatures3DWmrg | joint variance | 0.88 | 0.07 | 0.76 | 0.14 | 0.85 | 0.04 | 0.49 | 0.11 |
| glcmFeatures3DWmrg | joint entropy | 0.94 | 0.01 | 0.93 | 0.01 | 0.89 | 0.04 | 0.71 | 0.04 |
| glcmFeatures3DWmrg | difference average | 0.91 | 0.04 | 0.84 | 0.06 | 0.91 | 0.02 | 0.71 | 0.06 |
| glcmFeatures3DWmrg | difference variance | 0.82 | 0.09 | 0.67 | 0.18 | 0.83 | 0.05 | 0.49 | 0.13 |
| glcmFeatures3DWmrg | difference entropy | 0.94 | 0.02 | 0.91 | 0.02 | 0.91 | 0.03 | 0.72 | 0.03 |
| glcmFeatures3DWmrg | sum average | 0.96 | 0.02 | 0.92 | 0.03 | 0.96 | 0.01 | 0.9 | 0.03 |
| glcmFeatures3DWmrg | sum variance | 0.88 | 0.07 | 0.76 | 0.14 | 0.84 | 0.04 | 0.48 | 0.11 |
| glcmFeatures3DWmrg | sum entropy | 0.91 | 0.02 | 0.91 | 0.02 | 0.87 | 0.06 | 0.68 | 0.05 |
| glcmFeatures3DWmrg | angular second moment | 0.85 | 0.01 | 0.73 | 0 | 0.87 | 0.05 | 0.68 | 0.08 |
| glcmFeatures3DWmrg | contrast | 0.83 | 0.08 | 0.69 | 0.16 | 0.85 | 0.04 | 0.56 | 0.14 |
| glcmFeatures3DWmrg | dissimilarity | 0.91 | 0.04 | 0.84 | 0.06 | 0.91 | 0.02 | 0.71 | 0.06 |
| glcmFeatures3DWmrg | inverse difference | 0.94 | 0.01 | 0.92 | 0.01 | 0.92 | 0.03 | 0.76 | 0.03 |
| glcmFeatures3DWmrg | inverse difference normalised | 0.92 | 0.01 | 0.84 | 0.02 | 0.89 | 0.01 | 0.81 | 0.07 |
| glcmFeatures3DWmrg | inverse difference moment | 0.94 | 0.01 | 0.92 | 0.01 | 0.91 | 0.03 | 0.75 | 0.03 |
| glcmFeatures3DWmrg | inverse difference moment normalised | 0.91 | 0.02 | 0.82 | 0.03 | 0.88 | 0.01 | 0.8 | 0.06 |
| glcmFeatures3DWmrg | inverse variance | 0.93 | 0.01 | 0.93 | 0.01 | 0.9 | 0.03 | 0.75 | 0.03 |
| glcmFeatures3DWmrg | correlation | 0.76 | 0.05 | 0.71 | 0.04 | 0.8 | 0.07 | 0.65 | 0.06 |
| glcmFeatures3DWmrg | autocorrelation | 0.94 | 0.04 | 0.87 | 0.08 | 0.95 | 0.02 | 0.84 | 0.08 |
| glcmFeatures3DWmrg | cluster tendency | 0.88 | 0.07 | 0.76 | 0.14 | 0.84 | 0.04 | 0.48 | 0.11 |
| glcmFeatures3DWmrg | cluster shade | 0.47 | 0.28 | 0.46 | 0.24 | 0.49 | 0.14 | 0.14 | 0.1 |
| glcmFeatures3DWmrg | cluster prominence | 0.7 | 0.14 | 0.55 | 0.23 | 0.66 | 0.16 | 0.26 | 0.17 |
| glcmFeatures3DWmrg | first measure of information correlation | 0.81 | 0.03 | 0.66 | 0.04 | 0.84 | 0.03 | 0.68 | 0.03 |
| glcmFeatures3DWmrg | second measure of information correlation | 0.62 | 0.06 | 0.64 | 0.06 | 0.72 | 0.13 | 0.53 | 0.06 |
| GLRLMFeatures2Davg | short run emphasis | 0.91 | 0.01 | 0.87 | 0.01 | 0.89 | 0.01 | 0.72 | 0.03 |
| GLRLMFeatures2Davg | long runs emphasis | 0.92 | 0.01 | 0.86 | 0.01 | 0.88 | 0.01 | 0.7 | 0.04 |
| GLRLMFeatures2Davg | Low grey level run emphasis | 0.96 | 0.02 | 0.83 | 0.01 | 0.91 | 0.03 | 0.65 | 0.1 |
| GLRLMFeatures2Davg | High grey level run emphasis | 0.95 | 0.03 | 0.89 | 0.07 | 0.95 | 0.02 | 0.84 | 0.06 |
| GLRLMFeatures2Davg | Short run low grey level emphasis | 0.97 | 0.02 | 0.86 | 0.01 | 0.94 | 0.02 | 0.63 | 0.08 |
| GLRLMFeatures2Davg | Short run high grey level emphasis | 0.95 | 0.03 | 0.88 | 0.07 | 0.95 | 0.02 | 0.83 | 0.07 |
| GLRLMFeatures2Davg | Long run low grey level emphasis | 0.85 | 0.04 | 0.69 | 0.01 | 0.81 | 0.07 | 0.67 | 0.16 |
| GLRLMFeatures2Davg | Long run high grey level emphasis | 0.95 | 0.04 | 0.89 | 0.07 | 0.95 | 0.02 | 0.85 | 0.06 |
| GLRLMFeatures2Davg | Grey level non uniformity | 0.99 | 0 | 0.99 | 0 | 0.92 | 0.01 | 0.81 | 0.01 |
| GLRLMFeatures2Davg | Grey level non uniformity normalized | 0.88 | 0.02 | 0.91 | 0.01 | 0.85 | 0.02 | 0.69 | 0.03 |
| GLRLMFeatures2Davg | Run length non uniformity | 1 | 0 | 1 | 0 | 0.98 | 0 | 0.96 | 0 |
| GLRLMFeatures2Davg | Run length non uniformity normalized | 0.93 | 0.01 | 0.89 | 0.01 | 0.9 | 0.02 | 0.73 | 0.02 |
| GLRLMFeatures2Davg | Run percentage | 0.93 | 0.01 | 0.88 | 0.01 | 0.9 | 0.03 | 0.72 | 0.02 |
| GLRLMFeatures2Davg | Grey level variance | 0.85 | 0.07 | 0.75 | 0.12 | 0.78 | 0.05 | 0.38 | 0.09 |
| GLRLMFeatures2Davg | Run length variance | 0.95 | 0.01 | 0.84 | 0.02 | 0.9 | 0.02 | 0.69 | 0.05 |
| GLRLMFeatures2Davg | Run entropy | 0.93 | 0.01 | 0.96 | 0.01 | 0.88 | 0.04 | 0.7 | 0.02 |
| GLRLMFeatures2DDmrg | short run emphasis | 0.94 | 0.01 | 0.87 | 0.01 | 0.9 | 0.03 | 0.72 | 0.03 |
| GLRLMFeatures2DDmrg | long runs emphasis | 0.95 | 0.01 | 0.84 | 0.01 | 0.89 | 0.02 | 0.69 | 0.05 |
| GLRLMFeatures2DDmrg | Low grey level run emphasis | 0.96 | 0.03 | 0.84 | 0.01 | 0.93 | 0.02 | 0.61 | 0.09 |
| GLRLMFeatures2DDmrg | High grey level run emphasis | 0.95 | 0.04 | 0.88 | 0.07 | 0.95 | 0.02 | 0.83 | 0.08 |
| GLRLMFeatures2DDmrg | Short run low grey level emphasis | 0.96 | 0.02 | 0.87 | 0.01 | 0.94 | 0.01 | 0.6 | 0.06 |
| GLRLMFeatures2DDmrg | Short run high grey level emphasis | 0.95 | 0.04 | 0.88 | 0.07 | 0.95 | 0.02 | 0.83 | 0.08 |
| GLRLMFeatures2DDmrg | Long run low grey level emphasis | 0.93 | 0.05 | 0.68 | 0.01 | 0.89 | 0.02 | 0.63 | 0.14 |
| GLRLMFeatures2DDmrg | Long run high grey level emphasis | 0.94 | 0.04 | 0.88 | 0.07 | 0.95 | 0.02 | 0.84 | 0.08 |
| GLRLMFeatures2DDmrg | Grey level non uniformity | 1 | 0 | 1 | 0 | 0.95 | 0.01 | 0.88 | 0.01 |
| GLRLMFeatures2DDmrg | Grey level non uniformity normalized | 0.87 | 0.01 | 0.85 | 0.01 | 0.86 | 0.07 | 0.68 | 0.05 |
| GLRLMFeatures2DDmrg | Run length non uniformity | 1 | 0 | 1 | 0 | 0.98 | 0 | 0.96 | 0 |
| GLRLMFeatures2DDmrg | Run length non uniformity normalized | 0.94 | 0.01 | 0.89 | 0.01 | 0.9 | 0.03 | 0.73 | 0.02 |
| GLRLMFeatures2DDmrg | Run percentage | 0.94 | 0.01 | 0.88 | 0.01 | 0.9 | 0.03 | 0.73 | 0.03 |
| GLRLMFeatures2DDmrg | Grey level variance | 0.89 | 0.06 | 0.78 | 0.13 | 0.85 | 0.04 | 0.5 | 0.11 |
| GLRLMFeatures2DDmrg | Run length variance | 0.94 | 0.01 | 0.83 | 0.02 | 0.89 | 0.03 | 0.69 | 0.05 |
| GLRLMFeatures2DDmrg | Run entropy | 0.91 | 0.02 | 0.91 | 0.02 | 0.87 | 0.06 | 0.67 | 0.05 |
| GLRLMFeatures2DWmrg | short run emphasis | 0.92 | 0.01 | 0.87 | 0.01 | 0.89 | 0.02 | 0.71 | 0.03 |
| GLRLMFeatures2DWmrg | long runs emphasis | 0.93 | 0.01 | 0.85 | 0.01 | 0.88 | 0.02 | 0.7 | 0.04 |
| GLRLMFeatures2DWmrg | Low grey level run emphasis | 0.96 | 0.02 | 0.83 | 0.01 | 0.91 | 0.03 | 0.65 | 0.1 |
| GLRLMFeatures2DWmrg | High grey level run emphasis | 0.95 | 0.03 | 0.89 | 0.07 | 0.95 | 0.02 | 0.84 | 0.06 |
| GLRLMFeatures2DWmrg | Short run low grey level emphasis | 0.97 | 0.02 | 0.86 | 0.01 | 0.93 | 0.02 | 0.63 | 0.08 |
| GLRLMFeatures2DWmrg | Short run high grey level emphasis | 0.95 | 0.03 | 0.89 | 0.07 | 0.95 | 0.02 | 0.83 | 0.07 |
| GLRLMFeatures2DWmrg | Long run low grey level emphasis | 0.88 | 0.04 | 0.69 | 0.01 | 0.83 | 0.05 | 0.66 | 0.16 |
| GLRLMFeatures2DWmrg | Long run high grey level emphasis | 0.95 | 0.04 | 0.89 | 0.07 | 0.95 | 0.02 | 0.85 | 0.06 |
| GLRLMFeatures2DWmrg | Grey level non uniformity | 0.99 | 0 | 0.99 | 0 | 0.92 | 0.01 | 0.81 | 0.01 |
| GLRLMFeatures2DWmrg | Grey level non uniformity normalized | 0.88 | 0.02 | 0.9 | 0.01 | 0.85 | 0.02 | 0.68 | 0.03 |
| GLRLMFeatures2DWmrg | Run length non uniformity | 1 | 0 | 1 | 0 | 0.98 | 0 | 0.96 | 0 |
| GLRLMFeatures2DWmrg | Run length non uniformity normalized | 0.92 | 0.01 | 0.89 | 0.01 | 0.9 | 0.02 | 0.73 | 0.03 |
| GLRLMFeatures2DWmrg | Run percentage | 0.93 | 0.01 | 0.88 | 0.01 | 0.9 | 0.03 | 0.72 | 0.02 |
| GLRLMFeatures2DWmrg | Grey level variance | 0.85 | 0.07 | 0.75 | 0.12 | 0.78 | 0.05 | 0.38 | 0.09 |
| GLRLMFeatures2DWmrg | Run length variance | 0.94 | 0.01 | 0.85 | 0.02 | 0.89 | 0.02 | 0.7 | 0.05 |
| GLRLMFeatures2DWmrg | Run entropy | 0.93 | 0.02 | 0.96 | 0.01 | 0.86 | 0.05 | 0.67 | 0.02 |
| GLRLMFeatures2Dvmrg | short run emphasis | 0.94 | 0.01 | 0.86 | 0.01 | 0.9 | 0.03 | 0.72 | 0.03 |
| GLRLMFeatures2Dvmrg | long runs emphasis | 0.94 | 0.01 | 0.84 | 0.01 | 0.89 | 0.03 | 0.7 | 0.05 |
| GLRLMFeatures2Dvmrg | Low grey level run emphasis | 0.96 | 0.03 | 0.84 | 0.01 | 0.93 | 0.02 | 0.61 | 0.09 |
| GLRLMFeatures2Dvmrg | High grey level run emphasis | 0.95 | 0.04 | 0.88 | 0.07 | 0.95 | 0.02 | 0.83 | 0.08 |
| GLRLMFeatures2Dvmrg | Short run low grey level emphasis | 0.96 | 0.02 | 0.87 | 0.01 | 0.94 | 0.01 | 0.6 | 0.06 |
| GLRLMFeatures2Dvmrg | Short run high grey level emphasis | 0.95 | 0.04 | 0.88 | 0.07 | 0.95 | 0.02 | 0.83 | 0.08 |
| GLRLMFeatures2Dvmrg | Long run low grey level emphasis | 0.93 | 0.06 | 0.69 | 0.01 | 0.89 | 0.02 | 0.62 | 0.15 |
| GLRLMFeatures2Dvmrg | Long run high grey level emphasis | 0.94 | 0.04 | 0.88 | 0.07 | 0.95 | 0.02 | 0.85 | 0.08 |
| GLRLMFeatures2Dvmrg | Grey level non uniformity | 1 | 0 | 1 | 0 | 0.95 | 0.01 | 0.88 | 0.01 |
| GLRLMFeatures2Dvmrg | Grey level non uniformity normalized | 0.87 | 0.01 | 0.85 | 0.01 | 0.86 | 0.07 | 0.68 | 0.05 |
| GLRLMFeatures2Dvmrg | Run length non uniformity | 1 | 0 | 1 | 0 | 0.98 | 0 | 0.96 | 0 |
| GLRLMFeatures2Dvmrg | Run length non uniformity normalized | 0.94 | 0.01 | 0.89 | 0.01 | 0.9 | 0.03 | 0.73 | 0.02 |
| GLRLMFeatures2Dvmrg | Run percentage | 0.94 | 0.01 | 0.88 | 0.01 | 0.9 | 0.03 | 0.73 | 0.03 |
| GLRLMFeatures2Dvmrg | Grey level variance | 0.89 | 0.06 | 0.78 | 0.13 | 0.85 | 0.04 | 0.5 | 0.11 |
| GLRLMFeatures2Dvmrg | Run length variance | 0.94 | 0.01 | 0.83 | 0.02 | 0.88 | 0.03 | 0.69 | 0.05 |
| GLRLMFeatures2Dvmrg | Run entropy | 0.91 | 0.02 | 0.91 | 0.02 | 0.86 | 0.06 | 0.66 | 0.05 |
| GLRLMFeatures3Davg | short run emphasis | 0.92 | 0.01 | 0.87 | 0.01 | 0.9 | 0.03 | 0.72 | 0.04 |
| GLRLMFeatures3Davg | long runs emphasis | 0.9 | 0.01 | 0.83 | 0.01 | 0.89 | 0.02 | 0.7 | 0.06 |
| GLRLMFeatures3Davg | Low grey level run emphasis | 0.96 | 0.03 | 0.84 | 0.01 | 0.92 | 0.02 | 0.6 | 0.09 |
| GLRLMFeatures3Davg | High grey level run emphasis | 0.95 | 0.04 | 0.88 | 0.07 | 0.95 | 0.02 | 0.83 | 0.08 |
| GLRLMFeatures3Davg | Short run low grey level emphasis | 0.97 | 0.02 | 0.87 | 0.01 | 0.92 | 0.01 | 0.6 | 0.07 |
| GLRLMFeatures3Davg | Short run high grey level emphasis | 0.95 | 0.04 | 0.88 | 0.07 | 0.95 | 0.02 | 0.83 | 0.08 |
| GLRLMFeatures3Davg | Long run low grey level emphasis | 0.92 | 0.04 | 0.69 | 0.01 | 0.91 | 0.03 | 0.61 | 0.14 |
| GLRLMFeatures3Davg | Long run high grey level emphasis | 0.95 | 0.04 | 0.88 | 0.07 | 0.95 | 0.02 | 0.85 | 0.07 |
| GLRLMFeatures3Davg | Grey level non uniformity | 1 | 0 | 1 | 0 | 0.95 | 0.01 | 0.88 | 0.01 |
| GLRLMFeatures3Davg | Grey level non uniformity normalized | 0.88 | 0.01 | 0.85 | 0.01 | 0.86 | 0.07 | 0.69 | 0.05 |
| GLRLMFeatures3Davg | Run length non uniformity | 1 | 0 | 1 | 0 | 0.99 | 0 | 0.97 | 0 |
| GLRLMFeatures3Davg | Run length non uniformity normalized | 0.93 | 0.01 | 0.89 | 0.01 | 0.9 | 0.03 | 0.73 | 0.04 |
| GLRLMFeatures3Davg | Run percentage | 0.93 | 0.01 | 0.88 | 0.01 | 0.9 | 0.03 | 0.72 | 0.04 |
| GLRLMFeatures3Davg | Grey level variance | 0.89 | 0.06 | 0.78 | 0.13 | 0.85 | 0.04 | 0.5 | 0.11 |
| GLRLMFeatures3Davg | Run length variance | 0.9 | 0.01 | 0.83 | 0.01 | 0.88 | 0.03 | 0.7 | 0.06 |
| GLRLMFeatures3Davg | Run entropy | 0.92 | 0.02 | 0.92 | 0.02 | 0.87 | 0.06 | 0.68 | 0.05 |
| GLRLMFeatures3Dmrg | short run emphasis | 0.93 | 0.01 | 0.87 | 0.01 | 0.89 | 0.03 | 0.71 | 0.04 |
| GLRLMFeatures3Dmrg | long runs emphasis | 0.9 | 0.01 | 0.83 | 0.01 | 0.89 | 0.03 | 0.7 | 0.06 |
| GLRLMFeatures3Dmrg | Low grey level run emphasis | 0.96 | 0.03 | 0.84 | 0.01 | 0.92 | 0.02 | 0.6 | 0.09 |
| GLRLMFeatures3Dmrg | High grey level run emphasis | 0.95 | 0.04 | 0.88 | 0.07 | 0.95 | 0.02 | 0.83 | 0.08 |
| GLRLMFeatures3Dmrg | Short run low grey level emphasis | 0.97 | 0.03 | 0.87 | 0.01 | 0.92 | 0.02 | 0.6 | 0.08 |
| GLRLMFeatures3Dmrg | Short run high grey level emphasis | 0.95 | 0.04 | 0.88 | 0.07 | 0.95 | 0.02 | 0.83 | 0.08 |
| GLRLMFeatures3Dmrg | Long run low grey level emphasis | 0.92 | 0.04 | 0.7 | 0.01 | 0.91 | 0.03 | 0.61 | 0.14 |
| GLRLMFeatures3Dmrg | Long run high grey level emphasis | 0.95 | 0.04 | 0.88 | 0.07 | 0.95 | 0.02 | 0.85 | 0.07 |
| GLRLMFeatures3Dmrg | Grey level non uniformity | 1 | 0 | 1 | 0 | 0.95 | 0.01 | 0.88 | 0.01 |
| GLRLMFeatures3Dmrg | Grey level non uniformity normalized | 0.88 | 0.01 | 0.85 | 0.01 | 0.86 | 0.07 | 0.68 | 0.05 |
| GLRLMFeatures3Dmrg | Run length non uniformity | 1 | 0 | 1 | 0 | 0.99 | 0 | 0.97 | 0 |
| GLRLMFeatures3Dmrg | Run length non uniformity normalized | 0.93 | 0.01 | 0.89 | 0.01 | 0.9 | 0.03 | 0.73 | 0.04 |
| GLRLMFeatures3Dmrg | Run percentage | 0.93 | 0.01 | 0.88 | 0.01 | 0.9 | 0.03 | 0.72 | 0.04 |
| GLRLMFeatures3Dmrg | Grey level variance | 0.89 | 0.06 | 0.78 | 0.13 | 0.85 | 0.04 | 0.5 | 0.11 |
| GLRLMFeatures3Dmrg | Run length variance | 0.9 | 0.01 | 0.83 | 0.01 | 0.88 | 0.03 | 0.7 | 0.06 |
| GLRLMFeatures3Dmrg | Run entropy | 0.92 | 0.02 | 0.92 | 0.02 | 0.87 | 0.06 | 0.67 | 0.05 |
| GLSZMFeatures2Davg | small zone emphasis | 0.91 | 0.01 | 0.9 | 0.01 | 0.86 | 0.01 | 0.72 | 0.02 |
| GLSZMFeatures2Davg | Large zone emphasis | 0.9 | 0.04 | 0.73 | 0.03 | 0.81 | 0.02 | 0.63 | 0.07 |
| GLSZMFeatures2Davg | Low grey level zone emphasis | 0.96 | 0.03 | 0.83 | 0.01 | 0.91 | 0.01 | 0.73 | 0.11 |
| GLSZMFeatures2Davg | High grey level zone emphasis | 0.95 | 0.03 | 0.88 | 0.07 | 0.95 | 0.02 | 0.83 | 0.07 |
| GLSZMFeatures2Davg | Small zone low grey level emphasis | 0.82 | 0.02 | 0.92 | 0.01 | 0.88 | 0 | 0.78 | 0.06 |
| GLSZMFeatures2Davg | Small zone high grey level emphasis | 0.95 | 0.03 | 0.88 | 0.07 | 0.95 | 0.01 | 0.81 | 0.07 |
| GLSZMFeatures2Davg | Large zone low grey level emphasis | 0.74 | 0.16 | 0.36 | 0.01 | 0.77 | 0.04 | 0.58 | 0.11 |
| GLSZMFeatures2Davg | Large zone high grey level emphasis | 0.9 | 0.05 | 0.76 | 0.11 | 0.74 | 0.02 | 0.53 | 0.05 |
| GLSZMFeatures2Davg | Grey level non uniformity GLSZM | 0.99 | 0 | 0.99 | 0 | 0.98 | 0.01 | 0.92 | 0.01 |
| GLSZMFeatures2Davg | Grey level non uniformity normalized GLSZM | 0.87 | 0.02 | 0.91 | 0.01 | 0.83 | 0.02 | 0.67 | 0.03 |
| GLSZMFeatures2Davg | Zone size non uniformity | 0.99 | 0 | 1 | 0 | 0.82 | 0.04 | 0.6 | 0.03 |
| GLSZMFeatures2Davg | Zone size non uniformity normalized | 0.94 | 0.01 | 0.92 | 0.02 | 0.91 | 0.01 | 0.76 | 0.02 |
| GLSZMFeatures2Davg | Zone percentage GLSZM | 0.93 | 0.01 | 0.92 | 0.01 | 0.9 | 0.02 | 0.74 | 0.02 |
| GLSZMFeatures2Davg | Grey level variance GLSZM | 0.85 | 0.07 | 0.75 | 0.12 | 0.78 | 0.05 | 0.37 | 0.09 |
| GLSZMFeatures2Davg | Zone size variance | 0.93 | 0.02 | 0.7 | 0.03 | 0.84 | 0.02 | 0.63 | 0.08 |
| GLSZMFeatures2Davg | Zone size entropy | 0.96 | 0.01 | 0.97 | 0.01 | 0.92 | 0.02 | 0.81 | 0.02 |
| GLSZMFeatures2Dvmrg | small zone emphasis | 0.93 | 0.01 | 0.89 | 0.01 | 0.88 | 0.02 | 0.74 | 0.02 |
| GLSZMFeatures2Dvmrg | Large zone emphasis | 0.91 | 0.02 | 0.71 | 0.03 | 0.83 | 0.02 | 0.64 | 0.08 |
| GLSZMFeatures2Dvmrg | Low grey level zone emphasis | 0.94 | 0.03 | 0.87 | 0.01 | 0.94 | 0.01 | 0.74 | 0.04 |
| GLSZMFeatures2Dvmrg | High grey level zone emphasis | 0.95 | 0.04 | 0.88 | 0.07 | 0.95 | 0.02 | 0.83 | 0.08 |
| GLSZMFeatures2Dvmrg | Small zone low grey level emphasis | 0.84 | 0.02 | 0.92 | 0.01 | 0.88 | 0.03 | 0.76 | 0.06 |
| GLSZMFeatures2Dvmrg | Small zone high grey level emphasis | 0.95 | 0.04 | 0.87 | 0.08 | 0.94 | 0.02 | 0.81 | 0.08 |
| GLSZMFeatures2Dvmrg | Large zone low grey level emphasis | 0.84 | 0.11 | 0.46 | 0.03 | 0.81 | 0.02 | 0.61 | 0.02 |
| GLSZMFeatures2Dvmrg | Large zone high grey level emphasis | 0.89 | 0.05 | 0.74 | 0.12 | 0.76 | 0.02 | 0.56 | 0.06 |
| GLSZMFeatures2Dvmrg | Grey level non uniformity GLSZM | 1 | 0 | 1 | 0 | 0.99 | 0.01 | 0.96 | 0.01 |
| GLSZMFeatures2Dvmrg | Grey level non uniformity normalized GLSZM | 0.87 | 0.02 | 0.86 | 0.01 | 0.83 | 0.08 | 0.67 | 0.05 |
| GLSZMFeatures2Dvmrg | Zone size non uniformity | 1 | 0 | 1 | 0 | 0.82 | 0.04 | 0.61 | 0.04 |
| GLSZMFeatures2Dvmrg | Zone size non uniformity normalized | 0.94 | 0.01 | 0.92 | 0.02 | 0.91 | 0.01 | 0.76 | 0.02 |
| GLSZMFeatures2Dvmrg | Zone percentage GLSZM | 0.94 | 0.01 | 0.91 | 0.01 | 0.9 | 0.03 | 0.74 | 0.02 |
| GLSZMFeatures2Dvmrg | Grey level variance GLSZM | 0.89 | 0.06 | 0.78 | 0.13 | 0.85 | 0.04 | 0.49 | 0.1 |
| GLSZMFeatures2Dvmrg | Zone size variance | 0.91 | 0.02 | 0.68 | 0.02 | 0.83 | 0.02 | 0.63 | 0.09 |
| GLSZMFeatures2Dvmrg | Zone size entropy | 0.93 | 0.02 | 0.9 | 0.02 | 0.86 | 0.04 | 0.73 | 0.06 |
| GLSZMFeatures3D | small zone emphasis | 0.9 | 0.02 | 0.87 | 0.02 | 0.86 | 0.03 | 0.71 | 0.04 |
| GLSZMFeatures3D | Large zone emphasis | 0.89 | 0.09 | 0.79 | 0.05 | 0.69 | 0.03 | 0.53 | 0.03 |
| GLSZMFeatures3D | Low grey level zone emphasis | 0.9 | 0.05 | 0.85 | 0.03 | 0.87 | 0.05 | 0.78 | 0.08 |
| GLSZMFeatures3D | High grey level zone emphasis | 0.94 | 0.04 | 0.88 | 0.07 | 0.93 | 0.02 | 0.79 | 0.07 |
| GLSZMFeatures3D | Small zone low grey level emphasis | 0.61 | 0.06 | 0.46 | 0.03 | 0.67 | 0.14 | 0.51 | 0.21 |
| GLSZMFeatures3D | Small zone high grey level emphasis | 0.92 | 0.05 | 0.85 | 0.09 | 0.91 | 0.03 | 0.73 | 0.09 |
| GLSZMFeatures3D | Large zone low grey level emphasis | 0.85 | 0.14 | 0.6 | 0.11 | 0.71 | 0.02 | 0.55 | 0.04 |
| GLSZMFeatures3D | Large zone high grey level emphasis | 0.87 | 0.11 | 0.83 | 0.05 | 0.66 | 0.03 | 0.52 | 0.02 |
| GLSZMFeatures3D | Grey level non uniformity GLSZM | 1 | 0 | 1 | 0 | 0.84 | 0.01 | 0.73 | 0.02 |
| GLSZMFeatures3D | Grey level non uniformity normalized GLSZM | 0.89 | 0.02 | 0.83 | 0.01 | 0.78 | 0.06 | 0.6 | 0.04 |
| GLSZMFeatures3D | Zone size non uniformity | 0.98 | 0 | 0.99 | 0 | 0.44 | 0.08 | 0.15 | 0.02 |
| GLSZMFeatures3D | Zone size non uniformity normalized | 0.93 | 0.02 | 0.89 | 0.03 | 0.89 | 0.03 | 0.72 | 0.05 |
| GLSZMFeatures3D | Zone percentage GLSZM | 0.96 | 0.01 | 0.95 | 0.01 | 0.92 | 0.01 | 0.76 | 0.03 |
| GLSZMFeatures3D | Grey level variance GLSZM | 0.88 | 0.06 | 0.77 | 0.14 | 0.84 | 0.04 | 0.47 | 0.1 |
| GLSZMFeatures3D | Zone size variance | 0.89 | 0.09 | 0.79 | 0.06 | 0.69 | 0.03 | 0.54 | 0.03 |
| GLSZMFeatures3D | Zone size entropy | 0.95 | 0.02 | 0.93 | 0.01 | 0.9 | 0.03 | 0.78 | 0.02 |
| ngtdmFeatures2avg | coarseness | 0.93 | 0.01 | 0.92 | 0.02 | 0.89 | 0.02 | 0.86 | 0.03 |
| ngtdmFeatures2avg | contrast | 0.8 | 0.07 | 0.52 | 0.08 | 0.82 | 0.06 | 0.38 | 0.22 |
| ngtdmFeatures2avg | busyness | 0.74 | 0.02 | 0.72 | 0.01 | 0.61 | 0.02 | 0.6 | 0.08 |
| ngtdmFeatures2avg | complexity | 0.87 | 0.07 | 0.8 | 0.15 | 0.79 | 0.03 | 0.39 | 0.07 |
| ngtdmFeatures2avg | strength | 0.8 | 0.08 | 0.77 | 0.08 | 0.72 | 0.09 | 0.33 | 0.07 |
| ngtdmFeatures2Dmrg | coarseness | 0.97 | 0.01 | 0.94 | 0.02 | 0.95 | 0.01 | 0.86 | 0.06 |
| ngtdmFeatures2Dmrg | contrast | 0.93 | 0.04 | 0.74 | 0.06 | 0.94 | 0.02 | 0.72 | 0.12 |
| ngtdmFeatures2Dmrg | busyness | 0.94 | 0.02 | 0.83 | 0.03 | 0.84 | 0.03 | 0.66 | 0.04 |
| ngtdmFeatures2Dmrg | complexity | 0.81 | 0.08 | 0.75 | 0.17 | 0.76 | 0.07 | 0.35 | 0.1 |
| ngtdmFeatures2Dmrg | strength | 0.8 | 0.11 | 0.68 | 0.12 | 0.79 | 0.08 | 0.42 | 0.14 |
| ngtdmFeatures3D | coarseness | 0.97 | 0.01 | 0.95 | 0.01 | 0.94 | 0.01 | 0.86 | 0.06 |
| ngtdmFeatures3D | contrast | 0.92 | 0.05 | 0.74 | 0.07 | 0.93 | 0.02 | 0.71 | 0.12 |
| ngtdmFeatures3D | busyness | 0.95 | 0.02 | 0.84 | 0.03 | 0.84 | 0.03 | 0.66 | 0.04 |
| ngtdmFeatures3D | complexity | 0.82 | 0.08 | 0.75 | 0.18 | 0.76 | 0.07 | 0.34 | 0.1 |
| ngtdmFeatures3D | strength | 0.79 | 0.11 | 0.69 | 0.11 | 0.79 | 0.09 | 0.42 | 0.14 |
| gldzmFeatures2Davg | small distance emphasis GLDZM | 0.93 | 0.01 | 0.92 | 0.01 | 0.9 | 0.04 | 0.77 | 0.02 |
| gldzmFeatures2Davg | Large distance emphasis GLDZM | 0.99 | 0 | 0.99 | 0 | 0.99 | 0 | 0.96 | 0.01 |
| gldzmFeatures2Davg | Low grey level zone emphasis GLDZM | 0.96 | 0.03 | 0.83 | 0.01 | 0.91 | 0.01 | 0.73 | 0.11 |
| gldzmFeatures2Davg | High grey level zone emphasis GLDZM | 0.95 | 0.03 | 0.88 | 0.07 | 0.95 | 0.02 | 0.83 | 0.07 |
| gldzmFeatures2Davg | Small distance low grey level emphasis GLDZM | 0.95 | 0.03 | 0.81 | 0.01 | 0.89 | 0.01 | 0.73 | 0.12 |
| gldzmFeatures2Davg | Small distance high grey level emphasis GLDZM | 0.96 | 0.02 | 0.89 | 0.05 | 0.95 | 0.02 | 0.82 | 0.07 |
| gldzmFeatures2Davg | Large distance low grey level emphasis GLDZM | 0.96 | 0.01 | 0.85 | 0.01 | 0.9 | 0.02 | 0.68 | 0.11 |
| gldzmFeatures2Davg | Large distance high grey level emphasis GLDZM | 0.97 | 0.01 | 0.94 | 0.04 | 0.85 | 0.02 | 0.7 | 0.06 |
| gldzmFeatures2Davg | Grey level non uniformity GLDZM | 0.99 | 0 | 0.99 | 0 | 0.98 | 0.01 | 0.92 | 0.01 |
| gldzmFeatures2Davg | Grey level non uniformity normalized GLDZM | 0.87 | 0.02 | 0.91 | 0.01 | 0.83 | 0.02 | 0.67 | 0.03 |
| gldzmFeatures2Davg | Zone distance non uniformity GLDZM | 1 | 0 | 1 | 0 | 0.97 | 0 | 0.93 | 0 |
| gldzmFeatures2Davg | Zone distance non uniformity normalized GLDZM | 0.91 | 0.02 | 0.89 | 0.01 | 0.89 | 0.05 | 0.75 | 0.03 |
| gldzmFeatures2Davg | Zone percentage GLDZM | 0.93 | 0.01 | 0.92 | 0.01 | 0.9 | 0.02 | 0.74 | 0.02 |
| gldzmFeatures2Davg | Grey level variance GLDZM | 0.59 | 0.09 | 0.6 | 0.03 | 0.5 | 0.04 | 0.23 | 0.14 |
| gldzmFeatures2Davg | Zone distance variance GLDZM | 0.99 | 0 | 1 | 0 | 0.99 | 0 | 0.98 | 0 |
| gldzmFeatures2Davg | Zone distance entropy GLDZM | 0.96 | 0.01 | 0.97 | 0 | 0.9 | 0.02 | 0.75 | 0.01 |
| gldzmFeatures2Dmrg | small distance emphasis GLDZM | 0.93 | 0.01 | 0.92 | 0.01 | 0.9 | 0.04 | 0.79 | 0.02 |
| gldzmFeatures2Dmrg | Large distance emphasis GLDZM | 1 | 0 | 1 | 0 | 0.99 | 0 | 0.98 | 0 |
| gldzmFeatures2Dmrg | Low grey level zone emphasis GLDZM | 0.94 | 0.03 | 0.87 | 0.01 | 0.94 | 0.01 | 0.74 | 0.04 |
| gldzmFeatures2Dmrg | High grey level zone emphasis GLDZM | 0.95 | 0.04 | 0.88 | 0.07 | 0.95 | 0.02 | 0.83 | 0.08 |
| gldzmFeatures2Dmrg | Small distance low grey level emphasis GLDZM | 0.94 | 0.03 | 0.85 | 0.01 | 0.92 | 0.01 | 0.74 | 0.04 |
| gldzmFeatures2Dmrg | Small distance high grey level emphasis GLDZM | 0.96 | 0.03 | 0.89 | 0.05 | 0.95 | 0.02 | 0.82 | 0.09 |
| gldzmFeatures2Dmrg | Large distance low grey level emphasis GLDZM | 0.96 | 0.01 | 0.88 | 0.02 | 0.91 | 0.02 | 0.72 | 0.1 |
| gldzmFeatures2Dmrg | Large distance high grey level emphasis GLDZM | 0.98 | 0.01 | 0.95 | 0.03 | 0.84 | 0.04 | 0.7 | 0.07 |
| gldzmFeatures2Dmrg | Grey level non uniformity GLDZM | 1 | 0 | 1 | 0 | 0.99 | 0.01 | 0.96 | 0.01 |
| gldzmFeatures2Dmrg | Grey level non uniformity normalized GLDZM | 0.87 | 0.02 | 0.86 | 0.01 | 0.83 | 0.08 | 0.67 | 0.05 |
| gldzmFeatures2Dmrg | Zone distance non uniformity GLDZM | 1 | 0 | 1 | 0 | 0.98 | 0 | 0.95 | 0 |
| gldzmFeatures2Dmrg | Zone distance non uniformity normalized GLDZM | 0.91 | 0.01 | 0.88 | 0.01 | 0.87 | 0.05 | 0.77 | 0.04 |
| gldzmFeatures2Dmrg | Zone percentage GLDZM | 0.94 | 0.01 | 0.91 | 0.01 | 0.9 | 0.03 | 0.74 | 0.02 |
| gldzmFeatures2Dmrg | Grey level variance GLDZM | 0.89 | 0.06 | 0.78 | 0.13 | 0.85 | 0.04 | 0.49 | 0.1 |
| gldzmFeatures2Dmrg | Zone distance variance GLDZM | 1 | 0 | 1 | 0 | 0.99 | 0 | 0.99 | 0 |
| gldzmFeatures2Dmrg | Zone distance entropy GLDZM | 0.94 | 0.01 | 0.94 | 0.01 | 0.88 | 0.05 | 0.7 | 0.03 |
| gldzmFeatures3D | small distance emphasis GLDZM | 0.93 | 0.02 | 0.91 | 0.01 | 0.87 | 0.02 | 0.74 | 0.02 |
| gldzmFeatures3D | Large distance emphasis GLDZM | 0.98 | 0 | 0.99 | 0 | 0.95 | 0.03 | 0.89 | 0.03 |
| gldzmFeatures3D | Low grey level zone emphasis GLDZM | 0.9 | 0.05 | 0.85 | 0.03 | 0.87 | 0.05 | 0.78 | 0.08 |
| gldzmFeatures3D | High grey level zone emphasis GLDZM | 0.94 | 0.04 | 0.88 | 0.07 | 0.93 | 0.02 | 0.79 | 0.07 |
| gldzmFeatures3D | Small distance low grey level emphasis GLDZM | 0.9 | 0.05 | 0.85 | 0.03 | 0.87 | 0.05 | 0.77 | 0.08 |
| gldzmFeatures3D | Small distance high grey level emphasis GLDZM | 0.95 | 0.03 | 0.89 | 0.05 | 0.94 | 0.03 | 0.79 | 0.08 |
| gldzmFeatures3D | Large distance low grey level emphasis GLDZM | 0.88 | 0.08 | 0.87 | 0.02 | 0.85 | 0.03 | 0.78 | 0.08 |
| gldzmFeatures3D | Large distance high grey level emphasis GLDZM | 0.94 | 0.03 | 0.92 | 0.04 | 0.82 | 0.1 | 0.63 | 0.11 |
| gldzmFeatures3D | Grey level non uniformity GLDZM | 1 | 0 | 1 | 0 | 0.84 | 0.01 | 0.73 | 0.02 |
| gldzmFeatures3D | Grey level non uniformity normalized GLDZM | 0.89 | 0.02 | 0.83 | 0.01 | 0.78 | 0.06 | 0.6 | 0.04 |
| gldzmFeatures3D | Zone distance non uniformity GLDZM | 1 | 0 | 1 | 0 | 0.77 | 0 | 0.54 | 0.01 |
| gldzmFeatures3D | Zone distance non uniformity normalized GLDZM | 0.9 | 0.03 | 0.87 | 0.02 | 0.83 | 0.02 | 0.7 | 0.03 |
| gldzmFeatures3D | Zone percentage GLDZM | 0.96 | 0.01 | 0.95 | 0.01 | 0.92 | 0.01 | 0.76 | 0.03 |
| gldzmFeatures3D | Grey level variance GLDZM | 0.88 | 0.06 | 0.77 | 0.14 | 0.84 | 0.04 | 0.47 | 0.1 |
| gldzmFeatures3D | Zone distance variance GLDZM | 0.98 | 0 | 0.99 | 0 | 0.96 | 0.01 | 0.93 | 0.01 |
| gldzmFeatures3D | Zone distance entropy GLDZM | 0.95 | 0.01 | 0.94 | 0.01 | 0.85 | 0.04 | 0.65 | 0.03 |
| ngldmFeatures2Davg | Low dependence emphasis | 0.93 | 0.01 | 0.92 | 0.01 | 0.91 | 0.01 | 0.75 | 0.02 |
| ngldmFeatures2Davg | High dependence emphasis | 0.93 | 0.01 | 0.85 | 0.01 | 0.89 | 0.03 | 0.71 | 0.04 |
| ngldmFeatures2Davg | Low grey level count emphasis | 0.95 | 0.03 | 0.82 | 0.01 | 0.9 | 0.05 | 0.6 | 0.1 |
| ngldmFeatures2Davg | High grey level count emphasis | 0.95 | 0.03 | 0.89 | 0.07 | 0.95 | 0.02 | 0.84 | 0.06 |
| ngldmFeatures2Davg | Low dependence low grey level emphasis | 0.79 | 0.02 | 0.9 | 0.02 | 0.82 | 0.07 | 0.58 | 0.06 |
| ngldmFeatures2Davg | Low dependence high grey level emphasis | 0.95 | 0.03 | 0.87 | 0.08 | 0.95 | 0.01 | 0.8 | 0.07 |
| ngldmFeatures2Davg | High dependence low grey level emphasis | 0.8 | 0.05 | 0.61 | 0.01 | 0.77 | 0.05 | 0.63 | 0.16 |
| ngldmFeatures2Davg | High dependence high grey level emphasis | 0.89 | 0.07 | 0.87 | 0.08 | 0.9 | 0.01 | 0.8 | 0.05 |
| ngldmFeatures2Davg | Grey level non uniformity | 0.98 | 0 | 0.97 | 0 | 0.9 | 0.01 | 0.77 | 0.01 |
| ngldmFeatures2Davg | Grey level non uniformity normalized | 0.88 | 0.01 | 0.89 | 0.01 | 0.86 | 0.01 | 0.7 | 0.03 |
| ngldmFeatures2Davg | Dependence count non uniformity | 1 | 0 | 1 | 0 | 0.95 | 0.01 | 0.86 | 0.02 |
| ngldmFeatures2Davg | Dependence count non uniformity normalized | 0.92 | 0.01 | 0.91 | 0.02 | 0.92 | 0.03 | 0.79 | 0.04 |
| ngldmFeatures2Davg | Grey level variance | 0.85 | 0.07 | 0.75 | 0.12 | 0.78 | 0.05 | 0.38 | 0.09 |
| ngldmFeatures2Davg | Dependence count variance | 0.89 | 0.01 | 0.79 | 0.03 | 0.89 | 0.04 | 0.71 | 0.06 |
| ngldmFeatures2Davg | Dependence count entropy | 0.98 | 0 | 0.98 | 0 | 0.94 | 0.01 | 0.85 | 0.02 |
| ngldmFeatures2Davg | dependence Count Energy | 0.9 | 0.02 | 0.96 | 0.01 | 0.85 | 0.01 | 0.74 | 0.02 |
| ngldmFeatures2Dmrg | Low dependence emphasis | 0.94 | 0.01 | 0.92 | 0.01 | 0.91 | 0.02 | 0.76 | 0.02 |
| ngldmFeatures2Dmrg | High dependence emphasis | 0.94 | 0.01 | 0.84 | 0.01 | 0.89 | 0.03 | 0.71 | 0.04 |
| ngldmFeatures2Dmrg | Low grey level count emphasis | 0.96 | 0.03 | 0.82 | 0.01 | 0.92 | 0.03 | 0.56 | 0.1 |
| ngldmFeatures2Dmrg | High grey level count emphasis | 0.95 | 0.04 | 0.88 | 0.07 | 0.95 | 0.02 | 0.84 | 0.08 |
| ngldmFeatures2Dmrg | Low dependence low grey level emphasis | 0.84 | 0.02 | 0.91 | 0.02 | 0.79 | 0.12 | 0.46 | 0.08 |
| ngldmFeatures2Dmrg | Low dependence high grey level emphasis | 0.95 | 0.04 | 0.86 | 0.08 | 0.94 | 0.02 | 0.8 | 0.09 |
| ngldmFeatures2Dmrg | High dependence low grey level emphasis | 0.89 | 0.07 | 0.58 | 0.01 | 0.85 | 0.02 | 0.6 | 0.17 |
| ngldmFeatures2Dmrg | High dependence high grey level emphasis | 0.87 | 0.09 | 0.86 | 0.08 | 0.88 | 0.03 | 0.78 | 0.06 |
| ngldmFeatures2Dmrg | Grey level non uniformity | 0.99 | 0 | 1 | 0 | 0.93 | 0.01 | 0.86 | 0.01 |
| ngldmFeatures2Dmrg | Grey level non uniformity normalized | 0.88 | 0.01 | 0.84 | 0.01 | 0.87 | 0.06 | 0.69 | 0.05 |
| ngldmFeatures2Dmrg | Dependence count non uniformity | 1 | 0 | 1 | 0 | 0.95 | 0.01 | 0.86 | 0.02 |
| ngldmFeatures2Dmrg | Dependence count non uniformity normalized | 0.93 | 0.01 | 0.92 | 0.02 | 0.91 | 0.02 | 0.76 | 0.05 |
| ngldmFeatures2Dmrg | Grey level variance | 0.89 | 0.06 | 0.78 | 0.13 | 0.86 | 0.04 | 0.51 | 0.11 |
| ngldmFeatures2Dmrg | Dependence count variance | 0.91 | 0.01 | 0.82 | 0.02 | 0.86 | 0.03 | 0.7 | 0.05 |
| ngldmFeatures2Dmrg | Dependence count entropy | 0.92 | 0.02 | 0.91 | 0.02 | 0.87 | 0.03 | 0.72 | 0.05 |
| ngldmFeatures2Dmrg | dependence Count Energy | 0.84 | 0.02 | 0.83 | 0.02 | 0.83 | 0.05 | 0.69 | 0.05 |
| ngldmFeatures3Dmrg | Low dependence emphasis | 0.95 | 0.01 | 0.93 | 0.02 | 0.93 | 0.02 | 0.76 | 0.03 |
| ngldmFeatures3Dmrg | High dependence emphasis | 0.9 | 0.01 | 0.8 | 0.01 | 0.88 | 0.03 | 0.69 | 0.07 |
| ngldmFeatures3Dmrg | Low grey level count emphasis | 0.96 | 0.03 | 0.82 | 0.01 | 0.92 | 0.03 | 0.56 | 0.1 |
| ngldmFeatures3Dmrg | High grey level count emphasis | 0.95 | 0.04 | 0.88 | 0.07 | 0.95 | 0.02 | 0.84 | 0.08 |
| ngldmFeatures3Dmrg | Low dependence low grey level emphasis | 0.74 | 0.05 | 0.85 | 0.03 | 0.71 | 0.16 | 0.33 | 0.05 |
| ngldmFeatures3Dmrg | Low dependence high grey level emphasis | 0.91 | 0.06 | 0.83 | 0.11 | 0.91 | 0.04 | 0.72 | 0.11 |
| ngldmFeatures3Dmrg | High dependence low grey level emphasis | 0.86 | 0.05 | 0.45 | 0.01 | 0.88 | 0.01 | 0.57 | 0.13 |
| ngldmFeatures3Dmrg | High dependence high grey level emphasis | 0.86 | 0.06 | 0.82 | 0.08 | 0.77 | 0.02 | 0.54 | 0.03 |
| ngldmFeatures3Dmrg | Grey level non uniformity | 0.99 | 0 | 1 | 0 | 0.93 | 0.01 | 0.86 | 0.01 |
| ngldmFeatures3Dmrg | Grey level non uniformity normalized | 0.88 | 0.01 | 0.84 | 0.01 | 0.87 | 0.06 | 0.69 | 0.05 |
| ngldmFeatures3Dmrg | Dependence count non uniformity | 1 | 0 | 1 | 0 | 0.95 | 0.01 | 0.87 | 0.02 |
| ngldmFeatures3Dmrg | Dependence count non uniformity normalized | 0.93 | 0.02 | 0.9 | 0.03 | 0.92 | 0.02 | 0.78 | 0.03 |
| ngldmFeatures3Dmrg | Grey level variance | 0.89 | 0.06 | 0.78 | 0.13 | 0.86 | 0.04 | 0.51 | 0.11 |
| ngldmFeatures3Dmrg | Dependence count variance | 0.86 | 0.02 | 0.71 | 0.02 | 0.87 | 0.01 | 0.69 | 0.09 |
| ngldmFeatures3Dmrg | Dependence count entropy | 0.94 | 0.01 | 0.96 | 0.01 | 0.9 | 0.04 | 0.81 | 0.03 |
| ngldmFeatures3Dmrg | dependence Count Energy | 0.83 | 0.02 | 0.91 | 0.01 | 0.83 | 0.05 | 0.73 | 0.05 |

Note: The feature Dependence count percentage belonging to the family NGLDM (2Davg, 2Dmrg, 3Dmrg) was always 1 irrespective of the tumour. So, it is excluded in the analysis, which leaves 455 features.

Table S6. List of radiomic features with excellent mean ICC and SDM for S50p images

| **feature_group** | **feature_name** |
| --- | --- |
| Local intensity | local intensity peak |
| Local intensity | global intensity peak |
| Statistics | mean* |
| Statistics | median* |
| Statistics | 10th percentile |
| Statistics | 90th percentile |
| Statistics | Root mean |
| intensity volume | int at vol fraction 10 |
| Intensity histogram | mean* |
| Intensity histogram | median* |
| Intensity histogram | 10th percentile |
| Intensity histogram | 90th percentile |
| Intensity histogram | mode |
| glcmFeatures2Davg | joint maximum |
| glcmFeatures2Davg | joint average* |
| glcmFeatures2Davg | joint entropy |
| glcmFeatures2Davg | difference average |
| glcmFeatures2Davg | sum average* |
| glcmFeatures2Davg | dissimilarity |
| glcmFeatures2Davg | inverse difference |
| glcmFeatures2Davg | inverse difference moment |
| glcmFeatures2Davg | autocorrelation |
| glcmFeatures2DDmrg | joint maximum |
| glcmFeatures2DDmrg | joint average* |
| glcmFeatures2DDmrg | difference average |
| glcmFeatures2DDmrg | difference entropy |
| glcmFeatures2DDmrg | sum average* |
| glcmFeatures2DDmrg | dissimilarity |
| glcmFeatures2DDmrg | inverse difference |
| glcmFeatures2DDmrg | inverse difference moment |
| glcmFeatures2DDmrg | autocorrelation |
| glcmFeatures2Dmrg | joint average* |
| glcmFeatures2Dmrg | difference average |
| glcmFeatures2Dmrg | sum average* |
| glcmFeatures2Dmrg | dissimilarity |
| glcmFeatures2Dmrg | inverse difference |
| glcmFeatures2Dmrg | inverse difference moment |
| glcmFeatures2Dmrg | autocorrelation |
| glcmFeatures2Dvmrg | joint average* |
| glcmFeatures2Dvmrg | difference average |
| glcmFeatures2Dvmrg | difference entropy |
| glcmFeatures2Dvmrg | sum average* |
| glcmFeatures2Dvmrg | dissimilarity |
| glcmFeatures2Dvmrg | inverse difference |
| glcmFeatures2Dvmrg | inverse difference moment |
| glcmFeatures2Dvmrg | autocorrelation |
| glcmFeatures3Davg | joint average* |
| glcmFeatures3Davg | difference average |
| glcmFeatures3Davg | difference entropy |
| glcmFeatures3Davg | sum average* |
| glcmFeatures3Davg | dissimilarity |
| glcmFeatures3Davg | inverse difference |
| glcmFeatures3Davg | inverse difference normalised |
| glcmFeatures3Davg | inverse difference moment |
| glcmFeatures3Davg | inverse variance |
| glcmFeatures3Davg | autocorrelation |
| glcmFeatures3DWmrg | joint average* |
| glcmFeatures3DWmrg | difference average |
| glcmFeatures3DWmrg | difference entropy |
| glcmFeatures3DWmrg | sum average* |
| glcmFeatures3DWmrg | dissimilarity |
| glcmFeatures3DWmrg | inverse difference |
| glcmFeatures3DWmrg | inverse difference moment |
| glcmFeatures3DWmrg | inverse variance |
| glcmFeatures3DWmrg | autocorrelation |
| GLRLMFeatures2Davg | Low grey level run emphasis |
| GLRLMFeatures2Davg | High grey level run emphasis |
| GLRLMFeatures2Davg | Short-run low grey level emphasis |
| GLRLMFeatures2Davg | Short-run high grey level emphasis |
| GLRLMFeatures2Davg | Long run high grey level emphasis |
| GLRLMFeatures2Davg | Grey level non-uniformity |
| GLRLMFeatures2Davg | Run-length non-uniformity* |
| GLRLMFeatures2Davg | Run-length non-uniformity normalized |
| GLRLMFeatures2Davg | Run percentage |
| GLRLMFeatures2Davg | Run-length variance |
| GLRLMFeatures2DDmrg | Short-run emphasis |
| GLRLMFeatures2DDmrg | Low grey level run emphasis |
| GLRLMFeatures2DDmrg | High grey level run emphasis |
| GLRLMFeatures2DDmrg | Short-run low grey level emphasis |
| GLRLMFeatures2DDmrg | Short-run high grey level emphasis |
| GLRLMFeatures2DDmrg | Long run high grey level emphasis |
| GLRLMFeatures2DDmrg | Grey level non-uniformity |
| GLRLMFeatures2DDmrg | Run-length non-uniformity* |
| GLRLMFeatures2DDmrg | Run-length non-uniformity normalized |
| GLRLMFeatures2DDmrg | Run percentage |
| GLRLMFeatures2DWmrg | Low grey level run emphasis |
| GLRLMFeatures2DWmrg | High grey level run emphasis |
| GLRLMFeatures2DWmrg | Short-run low grey level emphasis |
| GLRLMFeatures2DWmrg | Short-run high grey level emphasis |
| GLRLMFeatures2DWmrg | Long run high grey level emphasis |
| GLRLMFeatures2DWmrg | Grey level non-uniformity |
| GLRLMFeatures2DWmrg | Run-length non-uniformity* |
| GLRLMFeatures2DWmrg | Run-length non-uniformity normalized |
| GLRLMFeatures2DWmrg | Run percentage |
| GLRLMFeatures2Dvmrg | Short-run emphasis |
| GLRLMFeatures2Dvmrg | Low grey level run emphasis |
| GLRLMFeatures2Dvmrg | High grey level run emphasis |
| GLRLMFeatures2Dvmrg | Short-run low grey level emphasis |
| GLRLMFeatures2Dvmrg | Short-run high grey level emphasis |
| GLRLMFeatures2Dvmrg | Long run high grey level emphasis |
| GLRLMFeatures2Dvmrg | Grey level non-uniformity |
| GLRLMFeatures2Dvmrg | Run-length -non-uniformity* |
| GLRLMFeatures2Dvmrg | Run-length non-uniformity normalized |
| GLRLMFeatures2Dvmrg | Run percentage |
| GLRLMFeatures3Davg | Short-run emphasis |
| GLRLMFeatures3Davg | Low grey level run emphasis |
| GLRLMFeatures3Davg | High grey level run emphasis |
| GLRLMFeatures3Davg | Short-run low grey level emphasis |
| GLRLMFeatures3Davg | Short-run high grey level emphasis |
| GLRLMFeatures3Davg | Long run low grey level emphasis |
| GLRLMFeatures3Davg | Long run high grey level emphasis |
| GLRLMFeatures3Davg | Grey level non-uniformity |
| GLRLMFeatures3Davg | Run-length non-uniformity* |
| GLRLMFeatures3Davg | Run-length non-uniformity normalized |
| GLRLMFeatures3Davg | Run percentage |
| GLRLMFeatures3Dmrg | Low grey level run emphasis |
| GLRLMFeatures3Dmrg | High grey level run emphasis |
| GLRLMFeatures3Dmrg | Short-run low grey level emphasis |
| GLRLMFeatures3Dmrg | Short-run high grey level emphasis |
| GLRLMFeatures3Dmrg | Long run low grey level emphasis |
| GLRLMFeatures3Dmrg | Long run high grey level emphasis |
| GLRLMFeatures3Dmrg | Grey level non-uniformity |
| GLRLMFeatures3Dmrg | Run-length non-uniformity* |
| GLRLMFeatures3Dmrg | Run-length non-uniformity normalized |
| GLRLMFeatures3Dmrg | Run percentage |
| GLSZMFeatures2Davg | Low grey-level zone emphasis |
| GLSZMFeatures2Davg | High grey-level zone emphasis |
| GLSZMFeatures2Davg | Small zone high grey level emphasis |
| GLSZMFeatures2Davg | Grey level non-uniformity GLSZM* |
| GLSZMFeatures2Davg | Zone size non-uniformity normalized |
| GLSZMFeatures2Davg | Zone percentage GLSZM |
| GLSZMFeatures2Davg | Zone size entropy |
| GLSZMFeatures2Dvmrg | Low grey-level zone emphasis |
| GLSZMFeatures2Dvmrg | High grey-level zone emphasis |
| GLSZMFeatures2Dvmrg | Small zone high grey level emphasis |
| GLSZMFeatures2Dvmrg | Grey level non-uniformity GLSZM* |
| GLSZMFeatures2Dvmrg | Zone size non-uniformity normalized |
| GLSZMFeatures2Dvmrg | Zone percentage GLSZM |
| GLSZMFeatures3D | High grey level zone emphasis |
| GLSZMFeatures3D | Small zone high grey level emphasis |
| GLSZMFeatures3D | Zone percentage GLSZM |
| GLSZMFeatures3D | Zone size entropy |
| ngtdmFeatures2Dmrg | coarseness |
| ngtdmFeatures2Dmrg | contrast |
| ngtdmFeatures3D | coarseness |
| ngtdmFeatures3D | contrast |
| gldzmFeatures2Davg | small distance emphasis GLDZM |
| gldzmFeatures2Davg | Large distance emphasis GLDZM* |
| gldzmFeatures2Davg | Low grey level zone emphasis GLDZM |
| gldzmFeatures2Davg | High grey level zone emphasis GLDZM |
| gldzmFeatures2Davg | Small distance high grey level emphasis GLDZM |
| gldzmFeatures2Davg | Large distance low grey level emphasis GLDZM |
| gldzmFeatures2Davg | Grey level non uniformity GLDZM* |
| gldzmFeatures2Davg | Zone distance non uniformity GLDZM* |
| gldzmFeatures2Davg | Zone percentage GLDZM |
| gldzmFeatures2Davg | Zone distance variance GLDZM* |
| gldzmFeatures2Davg | Zone distance entropy GLDZM |
| gldzmFeatures2Dmrg | small distance emphasis GLDZM |
| gldzmFeatures2Dmrg | Large distance emphasis GLDZM* |
| gldzmFeatures2Dmrg | Low grey level zone emphasis GLDZM |
| gldzmFeatures2Dmrg | High grey level zone emphasis GLDZM |
| gldzmFeatures2Dmrg | Small distance low grey level emphasis GLDZM |
| gldzmFeatures2Dmrg | Small distance high grey level emphasis GLDZM |
| gldzmFeatures2Dmrg | Large distance low grey level emphasis GLDZM |
| gldzmFeatures2Dmrg | Grey level non-uniformity GLDZM* |
| gldzmFeatures2Dmrg | Zone distance non-uniformity GLDZM* |
| gldzmFeatures2Dmrg | Zone percentage GLDZM |
| gldzmFeatures2Dmrg | Zone distance variance GLDZM* |
| gldzmFeatures3D | Large distance emphasis GLDZM |
| gldzmFeatures3D | High grey-level zone emphasis GLDZM |
| gldzmFeatures3D | Small distance high grey level emphasis GLDZM |
| gldzmFeatures3D | Zone percentage GLDZM |
| gldzmFeatures3D | Zone distance variance GLDZM* |
| ngldmFeatures2Davg | Low dependence emphasis |
| ngldmFeatures2Davg | Low grey level count emphasis |
| ngldmFeatures2Davg | High grey level count emphasis |
| ngldmFeatures2Davg | Low dependence high grey level emphasis |
| ngldmFeatures2Davg | Grey level non-uniformity |
| ngldmFeatures2Davg | Dependence count non-uniformity |
| ngldmFeatures2Davg | Dependence count non-uniformity normalized |
| ngldmFeatures2Davg | Dependence count entropy |
| ngldmFeatures2Dmrg | Low dependence emphasis |
| ngldmFeatures2Dmrg | Low grey level count emphasis |
| ngldmFeatures2Dmrg | High grey level count emphasis |
| ngldmFeatures2Dmrg | Low dependence high grey level emphasis |
| ngldmFeatures2Dmrg | Grey level non-uniformity |
| ngldmFeatures2Dmrg | Dependence count non-uniformity |
| ngldmFeatures2Dmrg | Dependence count non-uniformity normalized |
| ngldmFeatures3Dmrg | Low dependence emphasis |
| ngldmFeatures3Dmrg | Low grey level count emphasis |
| ngldmFeatures3Dmrg | High grey level count emphasis |
| ngldmFeatures3Dmrg | Low dependence high grey level emphasis |
| ngldmFeatures3Dmrg | Grey level non-uniformity |
| ngldmFeatures3Dmrg | Dependence count non-uniformity |
| ngldmFeatures3Dmrg | Dependence count non-uniformity normalized |
| ngldmFeatures3Dmrg | Dependence count entropy |

(* - Robust features for S25p images)


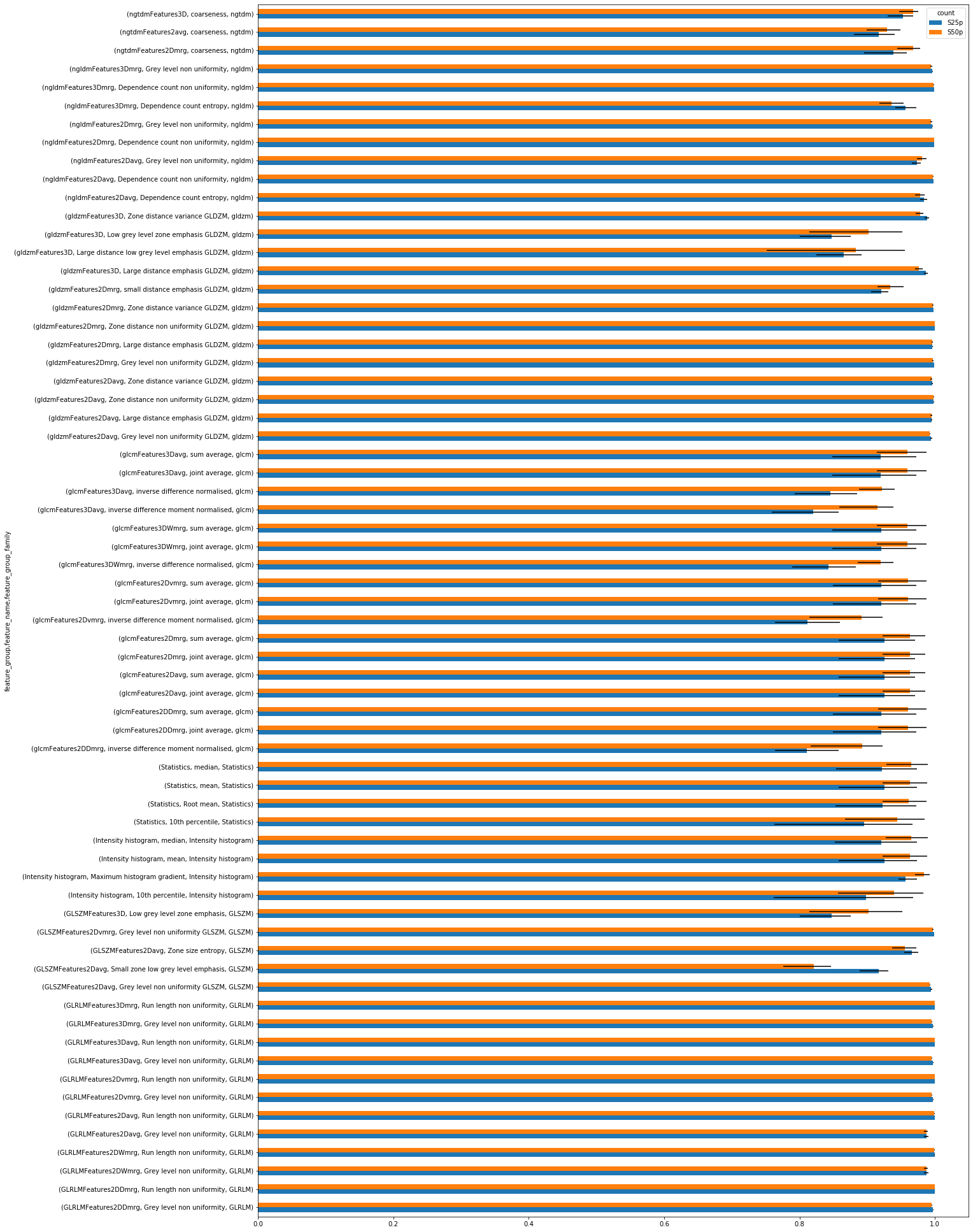


Fig. S3. ICC of features showing robustness to noise with good or moderate ICC and SDM. The error bars represent 95% CI for the mean bootstrapped ICC.


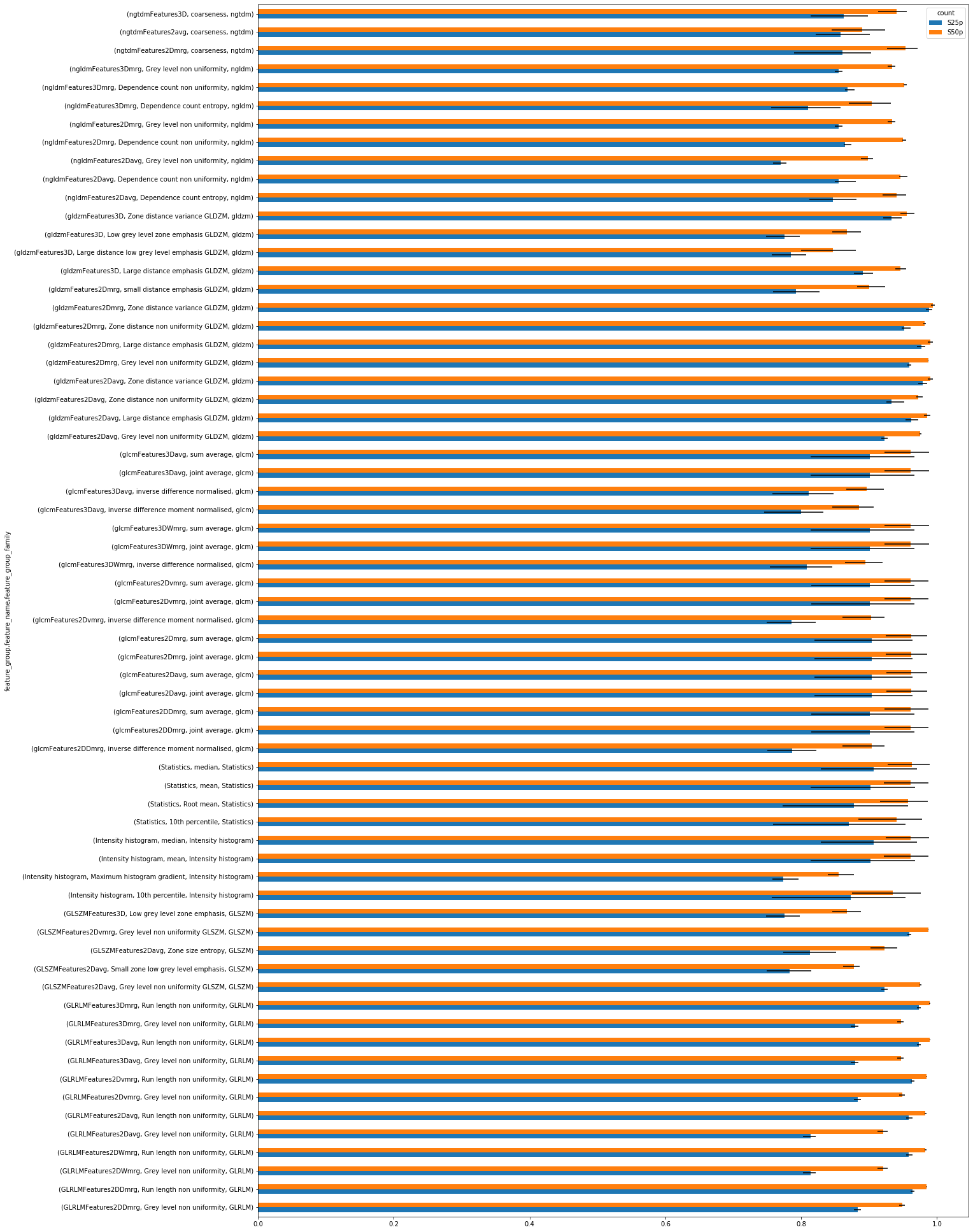


Fig. S4. SDM of features showing robustness to noise with good or moderate ICC and SDM. The error bars represent 95% CI for the mean bootstrapped SDM.
